# Endothelial Stat3 activation promotes osteoarthritis development

**DOI:** 10.1111/cpr.13518

**Published:** 2023-06-13

**Authors:** Jiadong Li, Wencai Zhang, Xinru Liu, Guangfeng Li, Yuyuan Gu, Kun Zhang, Fuming Shen, Xiang Wu, Yingying Jiang, Qin Zhang, Fengjin Zhou, Ke Xu, Jiacan Su

**Affiliations:** ^1^ Institute of Translational Medicine Shanghai University Shanghai China; ^2^ Organoid Research Center Shanghai University Shanghai China; ^3^ School of Medicine Shanghai University Shanghai China; ^4^ School of Life Sciences Shanghai University Shanghai China; ^5^ Department of Orthopedics, First Affiliated Hospital Jinan University Guangzhou China; ^6^ Department of Orthopedics Shanghai Zhongye Hospital Shanghai China; ^7^ Department of Orthopedics, Honghui Hospital Xi'an Jiao Tong University Xi'an China; ^8^ Wenzhou Institute of Shanghai University Wenzhou China; ^9^ Department of Orthopaedics Xinhua Hospital Affiliated to Shanghai Jiao Tong University School of Medicine Shanghai China

## Abstract

The mechanism of the balance between subchondral angiogenesis and articular damage within osteoarthritis (OA) progression remains a mystery. However, the lack of specific drugs leads to limited clinical treatment options for OA, frequently failing to prevent eventual joint destruction in patients. Increasing evidence suggests that subchondral bone angiogenesis precedes cartilage injury, while proliferating endothelial cells (ECs) induce abnormal bone formation. Signal transducer and activator of transcription 3 (Stat3) is triggered by multiple cytokines in the OA microenvironment. Here, we observed elevated Stat3 activation in subchondral bone H‐type vessels. Endothelial Stat3 activation will lead to stronger cell proliferation, migration and angiogenesis by simulating ECs in OA. In contrast, either Stat3 activation inhibition or knockdown of Stat3 expression could relieve such alterations. More interestingly, blocking Stat3 in ECs alleviated angiogenesis‐mediated osteogenic differentiation and chondrocyte lesions. Stat3 inhibitor reversed surgically induced subchondral bone H‐type vessel hyperplasia in vivo, significantly downregulating vessel volume and vessel number. Due to the reduced angiogenesis, subchondral bone deterioration and cartilage loss were alleviated. Overall, our data suggest that endothelial Stat3 activation is an essential trigger for OA development. Therefore, targeted Stat3 blockade is a novel promising therapeutic regimen for OA.

## Graphical abstract

Endothelial Stat3 is significantly activated by the co‐activation of multiple cytokines in the OA microenvironment. Endothelial Stat3 activation promotes osteoarthritic subchondral bone angiogenesis, which subsequently triggers bone deterioration and cartilage injury. Blocking endothelial Stat3 alleviated angiogenesis‐mediated osteogenic differentiation and chondrocyte lesions.

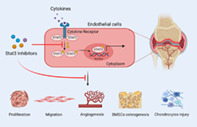

## 1 INTRODUCTION

Osteoarthritis (OA) is a widespread irreversible articular disease marked by articular cartilage deterioration and subsequent osteophytes.^1^, ^2^ It affects millions of people worldwide, especially older people over 60 years old.^3^ The current mainstream OA treatment options fall into three categories: physical therapy, pharmacotherapy and surgery.^4^, ^5^, ^6^ However, the different treatment options have their inherent drawbacks and the pathogenesis of OA is still unclear.^7^, ^8^ Therefore, finding more effective therapeutic strategies and targets for OA is of great significance. In normal knee joint tissue, from top to bottom, there is cartilage, calcified cartilage and subchondral bone sequentially.^9^, ^10^ When lesions occur, blood vessels with higher expression of CD31 and Endomucin (Emcn) are massively nascent in the subchondral bone.^11^ Previous studies have shown that blood vessels are closely associated with subchondral bone remodelling and nerve invasion.^12^, ^13^ During the progression of OA, these neovascularized endothelial cells (ECs) can induce cartilage degradation and recruit bone mesenchymal stem cells (BMSCs) for osteogenic differentiation.^14^ Eventually, in the late stages of OA, bottom‐up degradation of the cartilage matrix and subchondral osteosclerosis induced by vessels leads to severe joint stiffness. Meanwhile, nerves grow into the joint cavity along with blood vessels leading to pain.^14^, ^15^ This shows that blood vessels perform a crucial function in OA pathology.

Signal transducer and activator of transcription 3 (Stat3) is thought to be a common downstream transcriptional activator in response to multiple cytokines.^16^ In previous studies, Stat3 is thought to be a critical transcription factor regulating angiogenesis in a variety of diseases and tumours.^17^, ^18^, ^19^, ^20^ Endothelial Stat3 was activated directly or indirectly by enriched inflammatory factors (IL‐6, IL‐1β) and growth factors (VEGF, PDGF).^21^ Collectively, endothelial Stat3, co‐activated by above cytokines, may be the culprit of OA articular injury.

Here, we noted that endothelial Stat3 was activated in OA subchondral bone vessels to promote vascularization and articular lesions. Subsequently, proliferation, migration, and angiogenesis of ECs were suppressed by inhibiting Stat3 activation and knocking down Stat3 expression. More interestingly, inhibition of endothelial Stat3 alleviated ECs‐induced cartilage degradation and osteogenic differentiation of BMSCs. In vivo, Stat3 inhibition reverses angiogenesis and cartilage damage in subchondral bone of the destabilized medial meniscus (DMM) mice. It not only alleviated subchondral bone remodelling and sclerosis but also downregulated serum levels of inflammatory factors and angiogenic factors. Our findings provide new evidence for a pivotal role of endothelial Stat3 in neovascular‐mediated OA injury. It opens up new therapeutic strategies and targets for the treatment of OA.

## 2 MATERIALS AND METHODS

### 2.1 Cell

Mouse vascular endothelial cell lines (bEND.3) were purchased from KeyGEN BioTECH and cultured with Dulbecco's Modified Eagle Medium (DMEM, Corning). Mouse BMSCs w obtained from Cyagen Biosciences and cultured with Minimum Eagle Medium α (α‐MEM, Corning). Chondrocytes were extracted from the articular cartilage of newborn mice using type II collagenase (Worthington Biochemical) and cultured with DMEM/Nutrient Mixture F‐12 (DMEM/F12, Corning). All of the media were supplemented with 10% fetal bovine serum (FBS, Gibco), 100 U/mL penicillin (Gibco), and 100 μg/mL streptomycin (Gibco). All cells were cultured in a 5% CO_2_ atmosphere at 37°C with suitable humidity.

### 2.2 Cell viability assay

ECs (5 × 10^3^/well) were seeded in 96‐well plates and incubated for 24 h. Stattic (Selleck) dissolved in dimethyl sulfoxide (DMSO) was diluted in cell culture medium to a series of gradient concentrations (0.3125, 0.625, 1.25, 2.5, 5, 10 and 20 μM). The cells were subsequently transferred to different cell wells and incubated in a 37°C incubator for 48 h. After 48 h, cell viability was measured by the Cell Counting Kit‐8 (CCK‐8, Dojindo) kit according to the manufacturer's instructions. The absorbance at 450 nm was measured using a microplate reader (BioTek) after 1.5 h incubation at constant temperature.

### 2.3 Scratch wound assay

ECs were seeded in a 12‐well plate at a density of 2 × 10^5^/well. After confluence, the cells were scratched using a sterile pipette tip. The dead cells were washed off by PBS and then photographed for recording. ECs were incubated with IL‐6, IL‐6R and Stattic at 37°C for 48 h and then photographed to record again. The width of the wounded areas was calculated as healed wound area (%) = (*A*_0_ − *A*_48_)/*A*_0_ × 100, where *A*_0_ represents the initial wound area, and *A*_48_ represents the residual wound area at 48 h.

### 2.4 Transwell migration assay

ECs were incubated with IL‐6, IL‐6R and Stattic for 30 min before the migration assay. 2 × 10^4^ ECs in 300 μL of FBS‐free DMEM were added to the upper chamber (8.0 μm pore size, #353097, FALCON) and allowed to migrate towards 500 μL of complete DMEM. The unmigrated cells were removed from the upper chamber of the transwell chamber with a cotton swab after 24 h of incubation. Then the insert was fixed with 4% paraformaldehyde (PFA) for 10 min at room temperature. They were stained with 500 μL of 0.03% crystal violet solution for 30 min at 37°C. Finally, the chambers were washed three times in PBS for 3 min each time, waited for drying and then photographed and recorded under the microscope.

### 2.5 EdU proliferation assay

The EdU (5‐ethynyl‐2′‐deoxyuridine) proliferation kit (Beyotime) was used to investigate the proliferation of ECs. ECs were inoculated at 2 × 10^5^/well in 12‐well plates and incubated with 1× dilution of EdU for 2 h. They were subsequently fixed with PFA for 30 min and then permeabilized with PBS containing 0.3% Triton X‐100 (Beyotime) for 20 min. Each well was stained with click chemistry reaction solution and then observed under a fluorescence microscope at 594 nm. Hoechst was used to stain the nucleus.

### 2.6 Angiogenesis assay

ECs were incubated with IL‐6, IL‐6R and Stattic for 30 min before the angiogenesis assay. The bottom of the 96‐well plate was covered with 50uL of Matrigel (BD, #356234) and polymerized at 37°C for 30 min. Next, 2 × 10^4^ ECs were seeded into each well for incubation at 37°C in 5% CO_2_ for 4 h. ECs were photographed under a microscope.

### 2.7 Western blotting

Briefly, the cells were washed three times with PBS after removing the medium. RIPA lysis buffer (Beyotime) with protease inhibitor, phosphatase inhibitor and EDTA was then added. Cells were lysed on ice for 30 min and then the mixture was collected into a centrifuge tube using a cell spatula. The mixture was centrifuged at 12,000×*g* for 15 min. After that, it was quantified using the BCA protein quantification kit (Beyotime). Total proteins were separated in the SDS‐PAGE gel and transferred to the 0.45 μm immune‐blot PVDF membrane (Millipore). All membranes were blocked in TBST with 5% bovine serum albumin (BSA) for 2 h and incubated with primary antibody at 4°C overnight: Rabbit anti‐GAPDH (Abcam, ab181602, 1:10,000), Mouse anti‐Stat3 (CST, 9319, 1:1000), Rabbit anti‐p‐Stat3 (Abcam, ab76315, 1:10,000), Rabbit anti‐Collagen I (Abcam, ab270993, 1:1000), Rabbit anti‐RUNX2 (Abcam, ab92336, 1:5000), Rabbit anti‐OCN (Abcam, ab93876, 1:1000), Rabbit anti‐MMP3 (Abcam, ab52915, 1:5000), Rabbit anti‐MMP13 (Abcam, ab92336, 1:4000), Rabbit anti‐ Collagen II (Invitrogen, MA5‐12789, 1:1000). After trilateral washing with TBST, the membranes were further conjugated with Goat anti‐Rabbit IgG antibody (Abcam, ab6721, 1:3000) or Goat anti‐Mouse IgG antibody (Abcam, ab6789, 1:3000) for 1.5 h at room temperature. They were then visualized and imaged with a visual imaging system (Bio‐Rad). ImageJ software was used for semi‐quantification of western blotting bands.

### 2.8 Quantitative real‐time polymerase chain reaction

Total RNA was extracted from different cells using Trizol (Takara). The concentration of total RNA was quantified by measuring the absorbance at 260 nm. For SYBR Green‐based quantitative PCR amplification, the reaction was carried out in a volume of 20 μL with QTOWER (Analytik Jena). The reaction condition was 95°C for 3 min, following cycle 40 times at 95°C for 10 s, 60°C for 20 s, 72°C for 20 s and final 20 s at 72°. The 2^−ΔΔCt^ method was used to determine the relative expression level in different groups.

### 2.9 Collection of conditioned media

ECs were first incubated for 30 min in DMEM medium with 50 ng/mL IL‐6, 100 ng/mL IL‐6R and 2.5 μM Stattic. The supernatant was then removed and replaced with FBS‐free DMEM to further incubate at 37°C for 24 h. The supernatant was centrifuged at 1000 rpm for 5 min to remove the cells. The collected supernatant was supplemented with FBS to produce Stattic‐CM. Stattic‐CM was stored temporarily at 4°C for further use or −80°C for long‐term storage. After transfection of Stat3‐KD plasmid for 48 h, IL‐6 and IL‐6R were added to collect the Stat3‐KD‐CM. For IL‐6‐CM, Stattic (or Stat3‐KD plasmid) was replaced with an equal volume of DMSO (or blank plasmid).

### 2.10 Alkaline phosphatase and alizarin red staining

BMSCs were cultured with different conditioned media for 7 days and then washed once with PBS before being fixed with PFA. Subsequently, the BMSCs were washed again with PBS and stained with BCIP/NBT alkaline phosphatase colour development kit (Beyotime) at 37°C for 30 min. After completion of staining, BMSCs were washed three times with PBS for 5 min each time, and local or whole‐well imaging was performed using a microplate reader (BioTek). For alizarin red staining, BMSCs were stained with 2% alizarin red solution (Beyotime) after 21 days of incubation as described above.

### 2.11 Alcian Blue staining

Chondrocytes were cultured with different conditioned media for 48 h and then washed once with PBS before being fixed with PFA. Subsequently, the chondrocytes were washed once again with PBS and then stained with alcian blue solution (Sigma‐Aldrich) at 37°C for 30 min. After completion of staining, chondrocytes were washed three times with PBS for 5 min each time, and local or whole‐well imaging was performed using a microplate reader (BioTek).

### 2.12 Animal

All animal experiments were performed according to the guidelines evaluated and approved by the ethics committee of Shanghai University. To establish a stable OA model, the medial meniscus of the right knee was removed in C57BL/6 male mice. Subsequently, 10 mg/kg of Statttic and 20 mg/kg of Statttic were injected intraperitoneally three times a week into the experimental group. DMSO was injected in the same manner as a blank control. Mice were sacrificed 8 weeks after DMM modelling for histological analysis.

### 2.13 Von Frey test

Paw withdrawal thresholds were determined in mice according to the previously described.^13^ The animals were placed in a cage with a hollow grid at the bottom for 30 min to acclimate to the environment. Measurements were started when the mice remained calm. Needling was performed with Von Frey hair from below towards the plantar aspect of the surgical hind limb of the mice. As the force intensity increased, a positive response was recorded when the mice underwent a foot‐lifting movement. Each mouse was stimulated three times at 5 min intervals to take the average value and measured once a week before and after surgery.

### 2.14 Hind limb weight‐bearing test

The difference in hind limb weight bearing of mice was measured using a pressure sensing tester. Briefly, C57BL/6 mice were placed in a clear glass container. The container contains two pressure plates connected to electronic sensors. When the mice stand on the pressure plates with their hind limbs, the pressure difference between the left and right hind limbs is displayed on an electronic screen. The weight‐bearing difference in each group was measured before and 1, 2, 3, 4, 5, 6, 7 and 8 weeks after the DMM surgery (ΔWeight = normal hind limb − DMM hind limb).

### 2.15 Micro‐CT analysis

The joint tissues of the mice were fixed in 4% PFA for 24 h and the excess muscle tissue was removed. The entire knee joint is scanned using a microcomputed tomography system (Micro‐CT; Skyscan 1176, Bruker) for the portion of interest. Subsequently, 3D images of the sagittal plane of the medial tibial plateau were reconstructed and structural parameters of the subchondral bone were measured, including bone tissue volume/total tissue volume (BV/TV), subchondral bone plate thickness (SBP Th) and bone trabecular pattern factor (Tb.Pf).

### 2.16 Microangiography

After anaesthetising the mice with isoflurane (Sigma‐Aldrich), PBS with anticoagulants, 4% PFA and MicroFil MV‐120 (Flow‐Tech) were sequentially perfused through the heart into the vessels of the body circulation. Four millilitres of MV‐120 angiographic contrast agent was diluted with 5 mL of diluent and solidified with 0.5 mL of adhesive according to the manufacturer's instructions. The mixture needs to be used within 30 min. Tissue was soaked in 4% PFA after contrast infusion and stored at 4°C overnight for contrast agent polymerization. After 3 weeks of decalcification in EDTA solution, Micro‐CT scans and local revascularization were performed as described above.

### 2.17 Histology, immunochemistry and immunofluorescence analysis

Mice knee joints were fixed in 4% PFA and then immersed in 10% EDTA solution for decalcification for 3 weeks. Subsequently, all samples were embedded in paraffin. Sagittal sections of the medial knee were used for haematoxylin & eosin staining (H&E) and saffron & fixed green (S&F) staining. Meanwhile, OARSI scores were calculated as previously described.^22^ Immunohistochemistry and immunohistofluorescence were used to detect cartilage and subchondral bone changes. Knee sections of each group were immunostained by overnight incubation with antibodies to CD31 (Abcam, ab28364, 1:100), p‐Stat3 (Abcam, ab76315, 1:500), Emcn (Santa Cruz, V.7C7, 1:50), MMP13 (Abcam, ab219620, 1:300), Col‐2 (Abcam, ab34712, 1:200) and Osterix (Abcam, ab209484, 1:1000). DAPI was used to stain the nuclei of the cells. In addition, the safety of Stattic at short‐term application was evaluated by pathological staining of the heart, liver, spleen, lungs and kidneys.

### 2.18 ELISA

To check the expression levels of inflammatory factors (IL‐1β, IL‐6, TNF‐α) and pro‐vascular growth factors (VEGF, Ang‐2) in mice, serum samples from each group were isolated and then tested with ELISA kits (MultiSciences) according to the manufacturer's instructions. Concisely, whole blood was collected from mice and left to clot at room temperature, then centrifuged at 4000 rpm for 10 min to remove blood cells. Subsequently, 10 μL of serum and 90 μL of detection buffer were mixed and incubated in a 96‐well plate coated with the corresponding antibody, and finally, all samples were detected at 450 nm with a microplate reader.

### 2.19 Statistical analysis

All data are presented as mean ± standard deviation (SD). An unpaired *t*‐test was performed for comparisons between the two groups. Data analysis for three or more groups was performed by one‐way analysis of variance (ANOVA). The significance was accepted at *p < 0.05, **p < 0.01 and ***p < 0.001. GraphPad Prism 8 and Excel 2016 software were used for graphing and statistical analysis.

## 3 RESULTS

### 3.1 Single‐cell sequencing reveals alterations in subchondral bone ECs of OA patients

**FIGURE 1:**
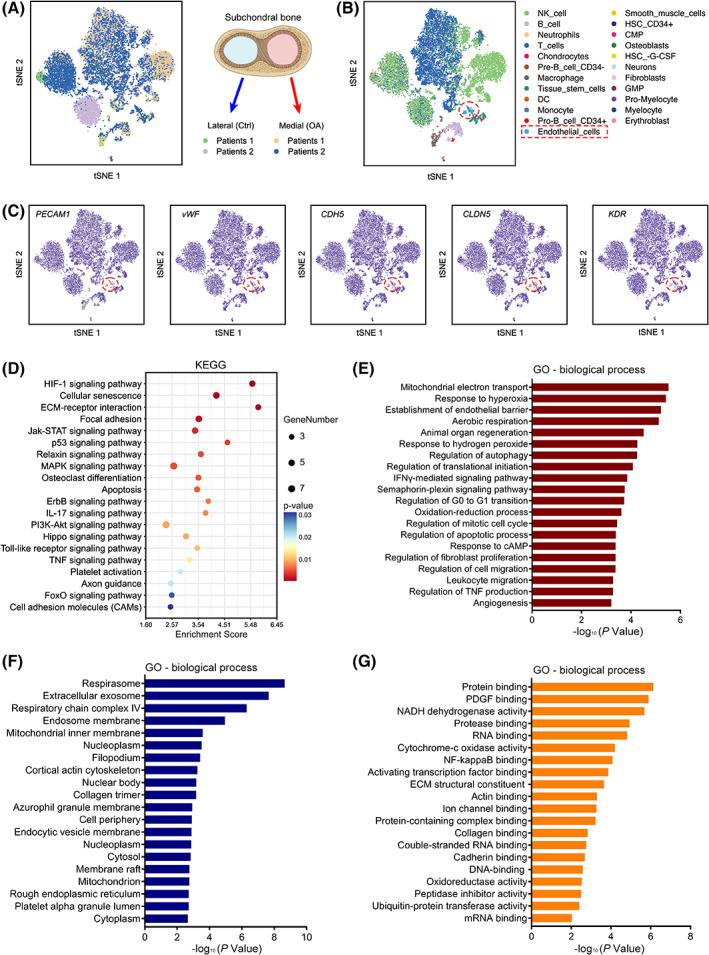
Single‐cell sequencing reveals alterations in human OA subchondral bone ECs. (A, B) The t‐stochastic neighbour embedding (tSNE) plots of all subchondral bone cells derived from four different sites. (C) Localization of endothelial cell marker expression on t‐SNE map. (D) KEGG pathway enrichment analysis of subchondral bone ECs in the OA and control groups. (E) GO functional enrichment analysis of heterogeneous biological process genes in subchondral bone ECs of control and OA groups. (F) GO functional enrichment analysis of heterogeneous cellular component genes in subchondral bone ECs of control and OA groups. (G) GO functional enrichment analysis of heterogeneous molecular function genes in subchondral bone ECs of control and OA groups.

Currently, subchondral bone deterioration is thought to precede the onset of cartilage damage.^23^, ^24^ Meanwhile, subchondral bone abnormal angiogenesis is observed in OA samples.^11^, ^25^ Therefore, we collected tibial plateaus from two OA patients to investigate the underlying mechanisms regulating subchondral bone ECs.^26^ We selected the medial tibial plateau where severe cartilage injury occurred as the OA group and the almost intact lateral plateau as the control group (Figure S1). The unbiased clustering of all cells isolated from the tibial plateau of two OA patients is divided into 23 clusters, and ECs were filtered by endothelial‐specific markers for the subsequent analysis (Figure 1A–C). To gain insight into the alterations of OA ECs, KEGG, and GO enrichment analysis was used to analyse the differences. Compared to the control group, OA ECs showed significant heterogeneity in the JAK–Stat pathway and inflammation‐related responses (Figure 1D). More interestingly, OA ECs showed more pronounced alterations in pro‐angiogenic properties, such as angiogenesis, cell migration, and endothelial barrier establishment (Figure 1E). Meanwhile, ECs in the OA microenvironment exhibited significant heterogeneity from controls in terms of secretory regulation and PDGF binding capacity (Figure 1F, G). As a result, OA ECs are more active in angiogenesis and possess stronger paracrine regulatory behaviour when regulated by multiple cytokines in the microenvironment.

### 3.2 Stat3 is activated in the subchondral bone H‐type vessels

**FIGURE 2:**
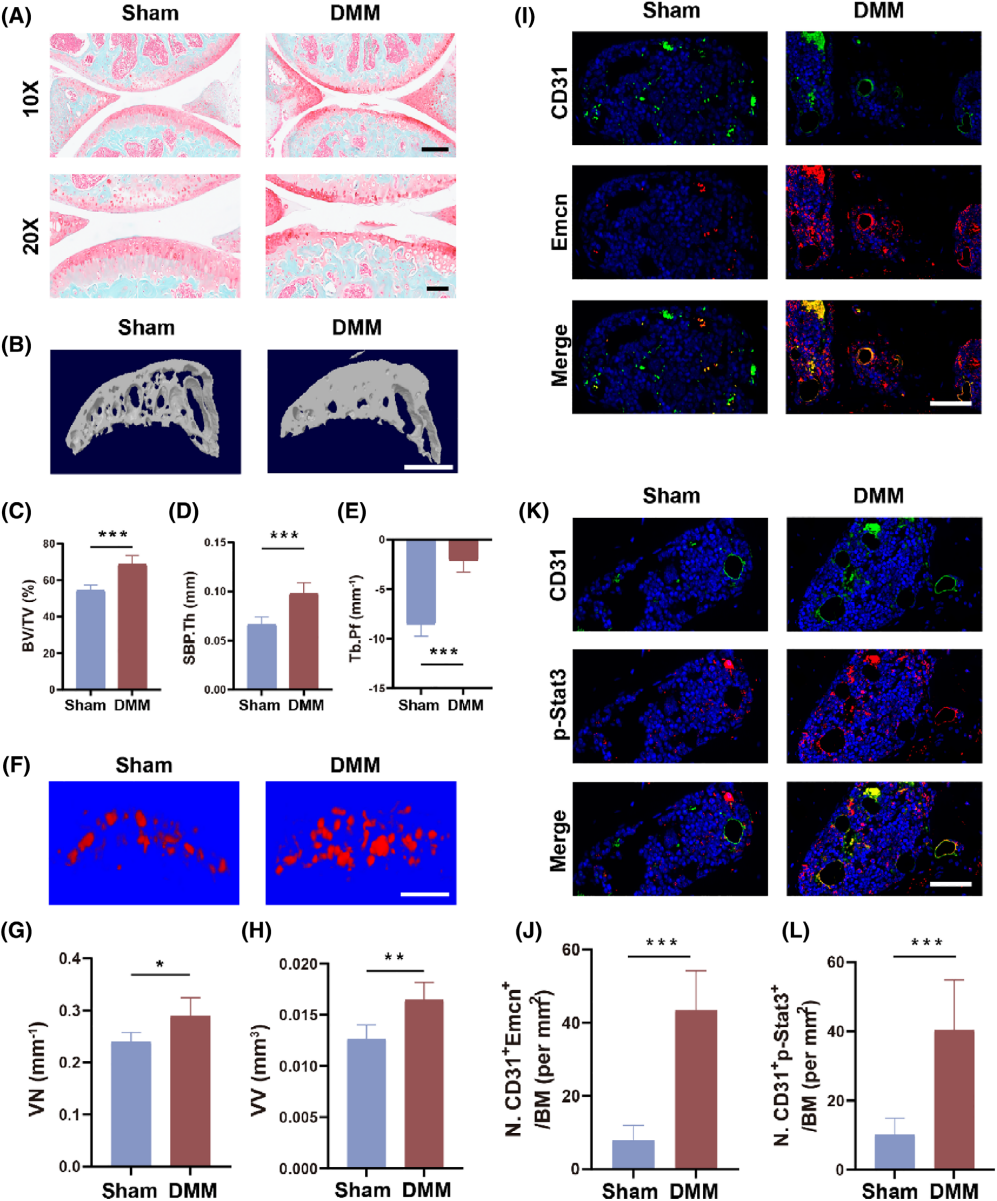
Stat3 is activated in the subchondral bone H‐type vessels. (A) S&F staining after 8 weeks of DMM modelling. Scale bar, 250 μm (10×). Scale bar, 100 μm (20×). (B) 3D reconstruction for medial tibial plateau subchondral bone sagittal plane 8 weeks after DMM. Scale bar, 500 μm. (C–E) Quantification of subchondral bone volume/tissue volume (BV/TV) (C), subchondral bone plate thickness (SBP.Th) (D), and trabecular separation (Tb.Pf) (E) in the medial tibial plateau by Micro‐CT. (F) 3D reconstruction for the sagittal plane of the medial subchondral bone blood vessels of the tibia by Micro‐CT at 8 weeks. Scale bar, 500 μm. (G, H) Quantification of vessel number (VN) (G) and vessel volume (VV) (H) in the Sham and DMM groups. (I, J) Representative immunofluorescence pictures (I) and quantitative analysis (J) of CD31 (green) and Emcn (red) positive cells. Scale bar, 50 μm. (K, L) Representative immunofluorescence pictures (K) and quantification (L) of CD31 (green) and p‐Stat3 (red) positive cells. Scale bar, 50 μm. All quantified data are presented with mean ± SD. The significance is represented as *p < 0.05, **p < 0.01 and ***p < 0.001.

Previous reports have suggested that vascular proliferation is a key factor in subchondral bone destruction, but the mechanism is unclear.^25^, ^27^ We destabilized the medial meniscus of the tibial plateau in mice to simulate the process of osteoarthritis (Figure S2). The saffron & fixed green (S&F) staining showed significant thinning of the cartilage layer and almost complete loss of hyaline cartilage (Figure 2A). Besides, severe osteophytes occurred in the subchondral bone of OA group (Figure 2B–E). Consistent with previous reports, we observed an abnormal increase in subchondral bone vessels (Figure 2F). A significant rise in vessel number and vessel volume occurred (Figure 2G,H). Similarly, immunohistofluorescence also showed a rise in CD31^+^ and Emcn^+^ H‐type vessels (Figure 2I,J). In Figure 1D, the JAK–Stat signalling pathway was significantly upregulated in OA patient ECs. Meanwhile, Stat3 is known to be intimately associated with pathological vascular proliferation.^19^, ^28^, ^29^ Notably, we noticed an elevated activation of Stat3 in H‐type vessels (Figure 2K). By immunofluorescent labelling, we found that phosphorylated Stat3 was localized in the neovascularization of subchondral bone (Figure 2L). Thus, we speculate that overactivated Stat3 in ECs may be a trigger for OA.

### 3.3 Inhibiting endothelial Stat3 activation alleviates OA‐induced angiogenesis

**FIGURE 3:**
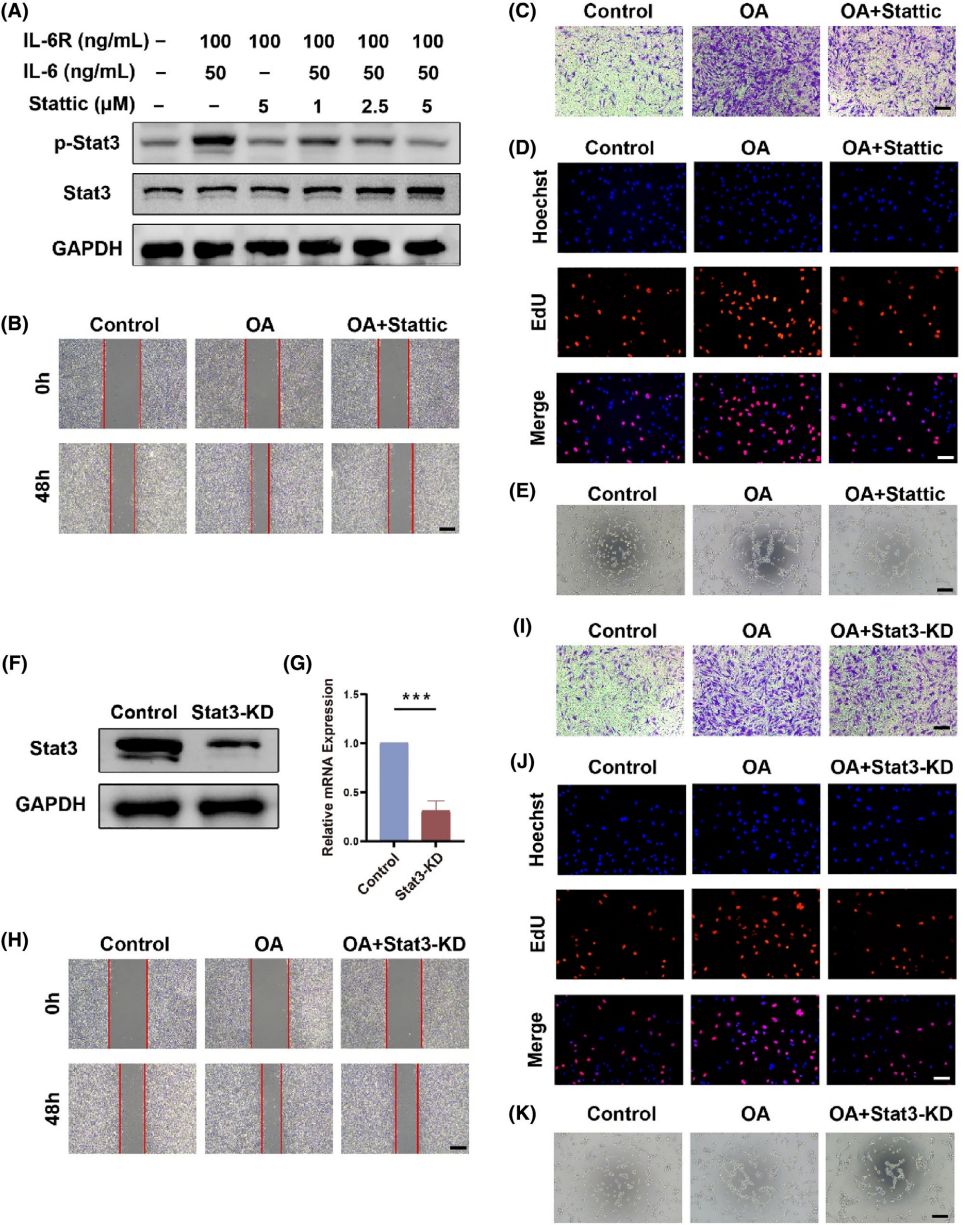
Inhibiting endothelial Stat3 activation alleviates OA‐induced angiogenesis. (A) Western blot images of Stattic inhibition of IL‐6/IL‐6R mediated endothelial Stat3 activation. (B) Wound healing examination of the migratory capacity in different groups of ECs. Scale bar, 200 μm. (C) Image of migrating cells from different groups shown by crystalline violet staining in transwell chambers. Scale bar, 200 μm. (D) EdU fluorescent labelling (red) assay detects the ratio of cells in proliferative phase. Hoechst (blue) labelled cell nucleus. Scale bar, 100 μm. (E) Tubes formed by ECs after 4 h incubation on matrigel. Scale bar, 200 μm. (F, G) Western blot images (F) and qRT‐PCR results (G) of ECs transfected with control plasmid and Stat3‐KD plasmid. (H) Wound healing examination of the migratory capacity in different groups of ECs. Scale bar, 200 μm. (I) Image of migrating cells from different groups shown by crystalline violet staining in transwell chambers. Scale bar, 200 μm. (J) EdU fluorescent labelling (red) assay detects the ratio of cells in proliferative phase. Hoechst (blue) labelled cell nucleus. Scale bar: 100 μm. (K) Tubes formed by ECs after 4 h incubation on matrigel. Scale bar, 200 μm. All quantified data are presented with mean ± SD. The significance is represented as *p < 0.05, **p < 0.01 and ***p < 0.001.

Due to complex OA micro‐environment, we first explored the possible activation capacity of Stat3 in ECs by adding different cells conditioned media and inflammatory factors. Among them, only preosteoclast‐conditioned media (POC‐CM) and IL‐6/IL‐6R significantly raised the level of p‐Stat3 (Figure S3). Considering the operability and stability, we chose IL‐6/IL‐6R as an activator of Stat3 to model vascular endothelial cells in OA. We explored the capability of IL‐6/IL‐6R to activate Stat3 of ECs (Figure S4). The addition of 50 ng/mL IL‐6 and 100 ng/mL IL‐6R was used as an OA‐induced angiogenesis model in subsequent experiments. When 2.5 μM of the specific inhibitor Stattic was added, the expression of p‐Stat3 in ECs was restored to normal levels without affecting normal proliferation (Figures 3A, S5 and S6). Also, the presence of IL‐6 or Stattic did not affect the expression of total Stat3. In Figure 3B,C, the migration capability of OA ECs is significantly improved. Inhibition of Stat3 activation effectively diminished the migratory capacity of ECs (Figure S7A,B). Meanwhile, by coupling red fluorescent thymidine EdU, ECs in a proliferative state exhibited a higher percentage of positivity under OA conditions (Figures 3D and S6C). Most importantly, the OA group formed more tubes and branching points on the Matrigel (Figure 3E). Stattic mitigates the mentioned changes, including the number of junctions, total tube length and total branch length (Figure S7D–F).

Besides reducing the phosphorylation activation of Stat3, the knockdown of total Stat3 could equally reflect the critical role of Stat3 in regulating OA‐induced angiogenesis. We knocked down the expression of Stat3 in ECs with plasmids, resulting in a significant reduction in Stat3 expression at the protein level and RNA level (Figure 3F,G and S8). When the expression of Stat3 was reduced, OA‐induced migration of ECs was significantly decreased (Figure 3H,I). The number of migrating cells and wound healing area in the visual field was reduced (Figure S7G,H). The number of red fluorescent‐positive cells in Figure 3J directly reflects that the knockdown of Stat3 downregulated the rise in proliferating cells. Thus, for OA‐induced proliferation of ECs, the knockdown of Stat3 remained significant (Figure S7I). Tube formation experiments similarly demonstrated the weakening of OA‐induced ECs' tube formation by knocking down Stat3 (Figure 3K). This change is characterized by a change in the junction number and total length (Figure S7J–L). To summarize, we successfully established an OA‐induced angiogenesis model and demonstrated that Stat3 activation is a key driver in regulating OA angiogenesis.

### 3.4 Endothelial Stat3 activation exacerbates angiogenesis‐related osteogenesis and cartilage degeneration

**FIGURE 4:**
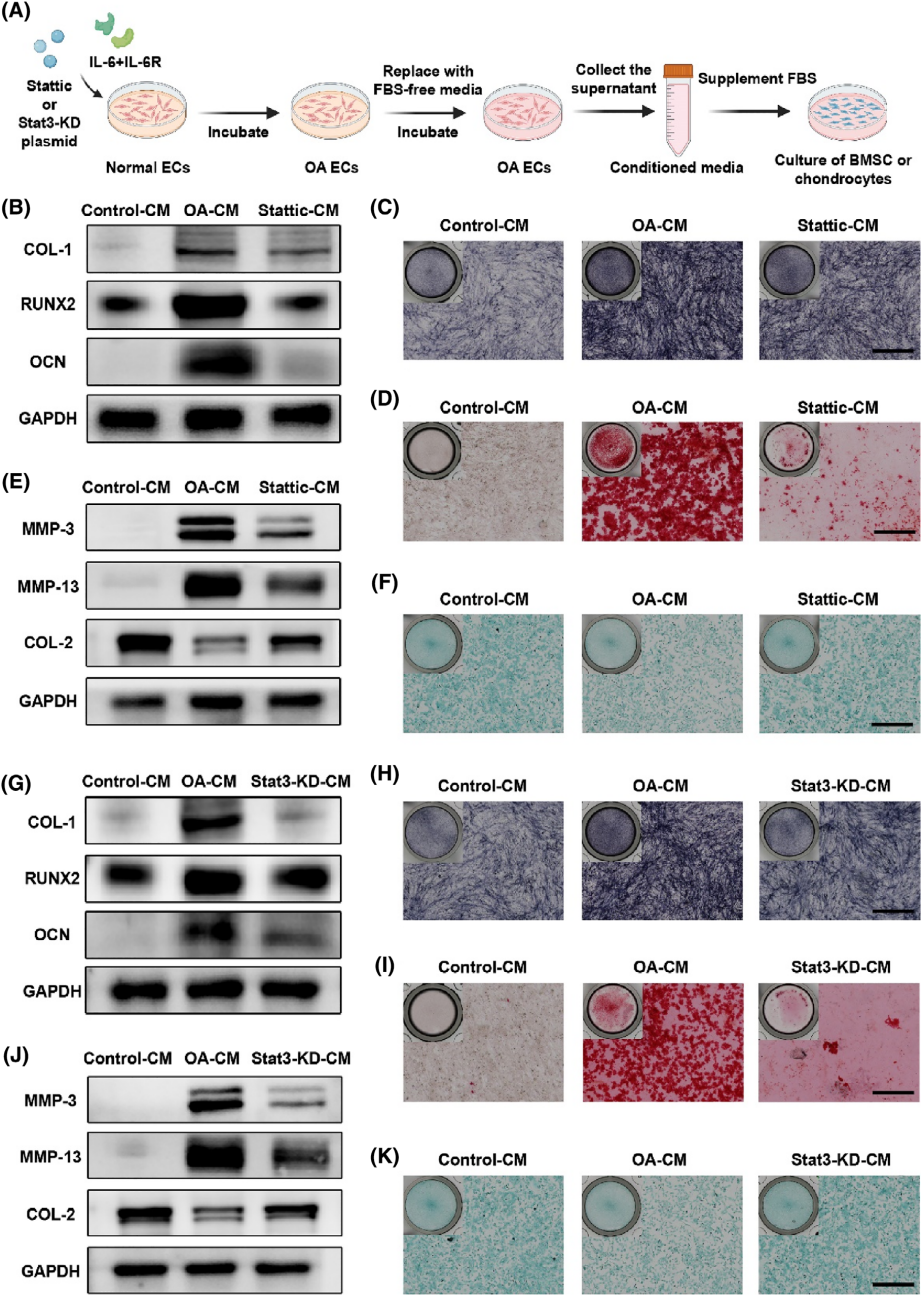
Endothelial Stat3 activation exacerbates angiogenesis‐related osteogenesis and cartilage degeneration. (A) Schematic diagrams of the different conditioned media were obtained. *Control*‐*CM*: supernatant of normal ECs. *OA*‐*CM*: supernatant of ECs after 30 min incubation with IL‐6/IL‐6R. *Stattic*‐*CM*: supernatant of ECs after 30 min co‐incubation with IL‐6/IL‐6R and Stattic. *Stat3*‐*KD*‐*CM*: supernatant of Stat3‐KD ECs after 30 min incubation with IL‐6/IL‐6R. (B) Western blot images of osteogenic markers (COL‐1, RUNX2, OCN) of BMSCs after 7 days incubation with conditioned medium. (C, D) Whole‐well and local images of BMSCs in 24‐well plates stained with alkaline phosphatase (7 days) (C) and alizarin red staining (21 days) (D). Scale bar, 500 μm. (E) Western blot images of chondrocyte catabolic markers (MMP‐3, MMP‐13) and anabolic markers (COL‐2) after 48 h of culture with conditioned medium. (F) Whole‐well and local images of alcian blue staining of chondrocytes after 48 h incubation with conditioned medium. Scale bar, 500 μm. (G) Western blot images of osteogenic markers (COL‐1, RUNX2, OCN) of BMSCs after 7 days incubation with conditioned medium. (H, I) Whole‐well and local images of BMSCs in 24‐well plates stained with alkaline phosphatase (7 days) (H) and alizarin red staining (21 days) (I). Scale bar, 500 μm. (J) Western blot images of chondrocyte catabolic markers (MMP‐3, MMP‐13) and anabolic markers (COL‐2) after 48 h of culture with conditioned medium. (K) Whole‐well and local images of alcian blue staining of chondrocytes after 48 h incubation with conditioned medium. Scale bar, 500 μm.

Previous studies have suggested that pathological vessels in OA subchondral bone bring more nutrients and oxygen, which in turn induce osteogenic differentiation of BMSCs and extracellular matrix lesions of chondrocytes.^30^ Moreover, there is a close correlation between ECs and MSCs, ECs and chondrocytes in the subchondral bone.^26^ In order to investigate how the endothelial Stat3 pathway works, we designed a research plan to simulate the in vivo OA microenvironment as shown in Figure 4A. With Stat3 activated, ECs induced higher transcription and translation of osteogenic markers (ALP, COL‐1, RUNX2, OCN) in BMSCs (Figures 4B,C and S9 A–D). This osteogenic differentiation was reversed when phosphorylation of Stat3 was downregulated by Stattic (Figure S10A–C). Similar findings were found in ALP staining (Figure 4C). In Figure 4D, BMSCs of the OA‐CM group produced massive calcium deposition after 14 days of culture, while the Stattic‐CM group produced only a slight amount of calcium minerals. In the articular joints of patients with OA, vascular invasion into the articular cartilage leading to cartilage destruction can be observed. Considering that, we examined the effect of ECs on chondrocytes. After incubation with OA‐CM for 48 h, MMP‐3 and MMP‐13 in chondrocytes were substantially increased, while collagen content was significantly reduced (Figures 4E and S9E–H). When Stat3 activation was inhibited, the erosive effect of OA ECs on chondrocytes was alleviated (Figure S10D–F). A similar trend was demonstrated by staining with Alcian blue, and chondrocytes in the Stattic‐CM group recovered the type II collagen content of the extracellular matrix (Figure 4F). More interestingly, OA‐CM invoked the secretion of IL‐6 and VEGF by chondrocytes, both of which can activate the Stat3 signalling pathway in ECs (Figure S9I,J). The addition of Stattic inhibited chondrocytes from promoting angiogenesis in the form of positive feedback.

Subsequently, we assessed the effects of knocking down ECs Stat3 on BMSCs and chondrocytes in a similar manner as described above. We noted that knockdown of Stat3 in ECs similarly reversed the process of osteo‐differentiation in BMSCs, most notably the reduction of COL‐1 (Figure 4G). All osteogenic markers, including OCN, were significantly decreased after the intervention of Stat3 expression (Figures S11A–D and S12A–C). Similarly, compared to OA‐CM, ALP and alizarin red staining reflected a decrease in osteogenic differentiated BMSCs in the Stat3‐KD‐CM group (Figure 4H,I). Western blot and alcian blue staining results showed that knockdown of Stat3 in ECs significantly saved chondrocytes lesion and restored the extracellular matrix (Figure 4J,K). Chondrocyte catabolic enzymes (MMP‐3, MMP‐13) and anabolic enzymes (COL‐2, Aggrecan) were significantly restored, indicating that vascular‐induced cartilage erosion was alleviated (Figures S11E–H and S12D–F). VEGF production in chondrocytes was restored to baseline levels by Stat3‐KD‐CM (Figure S11I). Transcription of the inflammatory factor IL‐6 was downregulated by a drop in Stat3 of ECs (Figure S11J). The above evidence suggests that over‐activated Stat3 in ECs will promote osteogenic differentiation of BMSCs and secrete calcium minerals to form bone, while inducing extracellular matrix breakdown in chondrocytes. Either blocking Stat3 phosphorylation or knocking down Stat3 could alleviate such alterations. This indicates that Stat3 is a critical mediator and driver of coupling angiogenesis, osteogenesis and cartilage injury in OA.

### 3.5 Blocking Stat3 attenuates OA joint injury and pain behaviour

**FIGURE 5:**
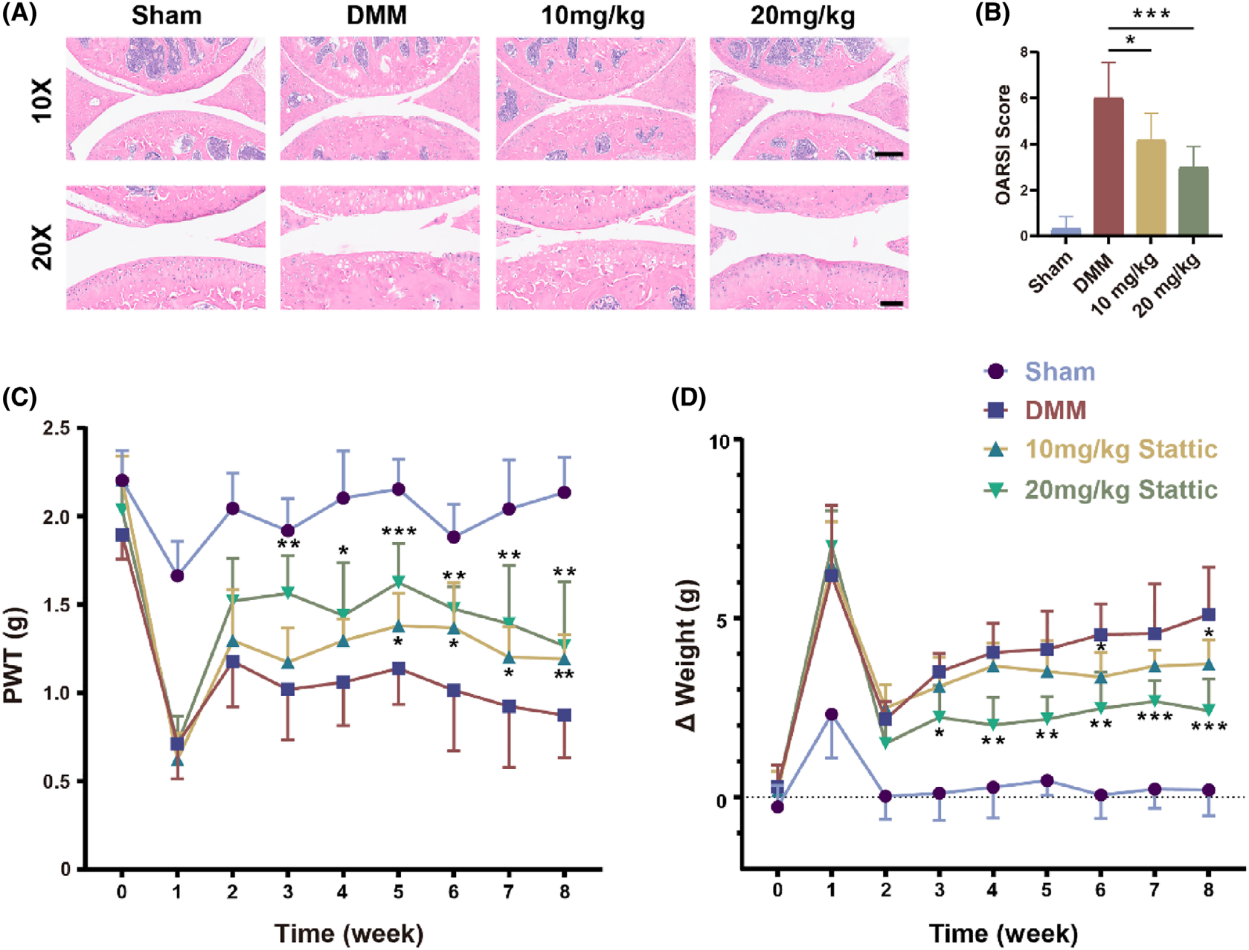
Blocking Stat3 attenuates OA joint injury and pain behaviour. (A) H&E stained images of the Stattic were administered intraperitoneally every 2 days. Scale bar, 250 μm (10×). Scale bar, 100 μm (20×). (B) OARSI histological scoring of articular cartilage of the tibial plateau in four groups after DMM surgery. (C, D) Pain‐related paw withdrawal thresholds (PWT) (C) and hind limb weight‐bearing (D) behavioural tests from 0 to 8 weeks. All quantified data are presented with mean ± SD. The significance is represented as *p < 0.05, **p < 0.01 and ***p < 0.001.

We evaluated the therapeutic effects on DMM model C57BL/6 mice through intraperitoneal injection of Stattic. After 8 weeks of intraperitoneal injection (three times a week), Stattic did not show any remarkable organ toxicity (Figure S13). As shown in Figure 5A, the structure of the articular cartilage of the mice was restored after 8 weeks of continuous administration. The OARSI score reflects the relief of arthritis in mice (Figure 5B). We evaluated the severity of OA and its effect on activity behaviour in mice by the von Frey test and the hind limb weight‐bearing test (Figure 5C,D). As OA progresses, the animal becomes more sensitive to stabbing pain and tends to perform body weight bearing to the non‐operative side. Stattic administration at 20 mg/kg significantly improved paw withdrawal thresholds from the third week while reducing weight‐bearing difference. However, 10 mg/kg did not show variability until the sixth week, probably due to most drugs being rapidly metabolized by the liver without reaching the joint. In conclusion, these data suggest that blocking Stat3 alleviates joint injury and pain in DMM mice, demonstrating its effectiveness for OA treatment.

### 3.6 Blocking Stat3 inhibits H‐type vessel neogenesis under OA inflammation

**FIGURE 6:**
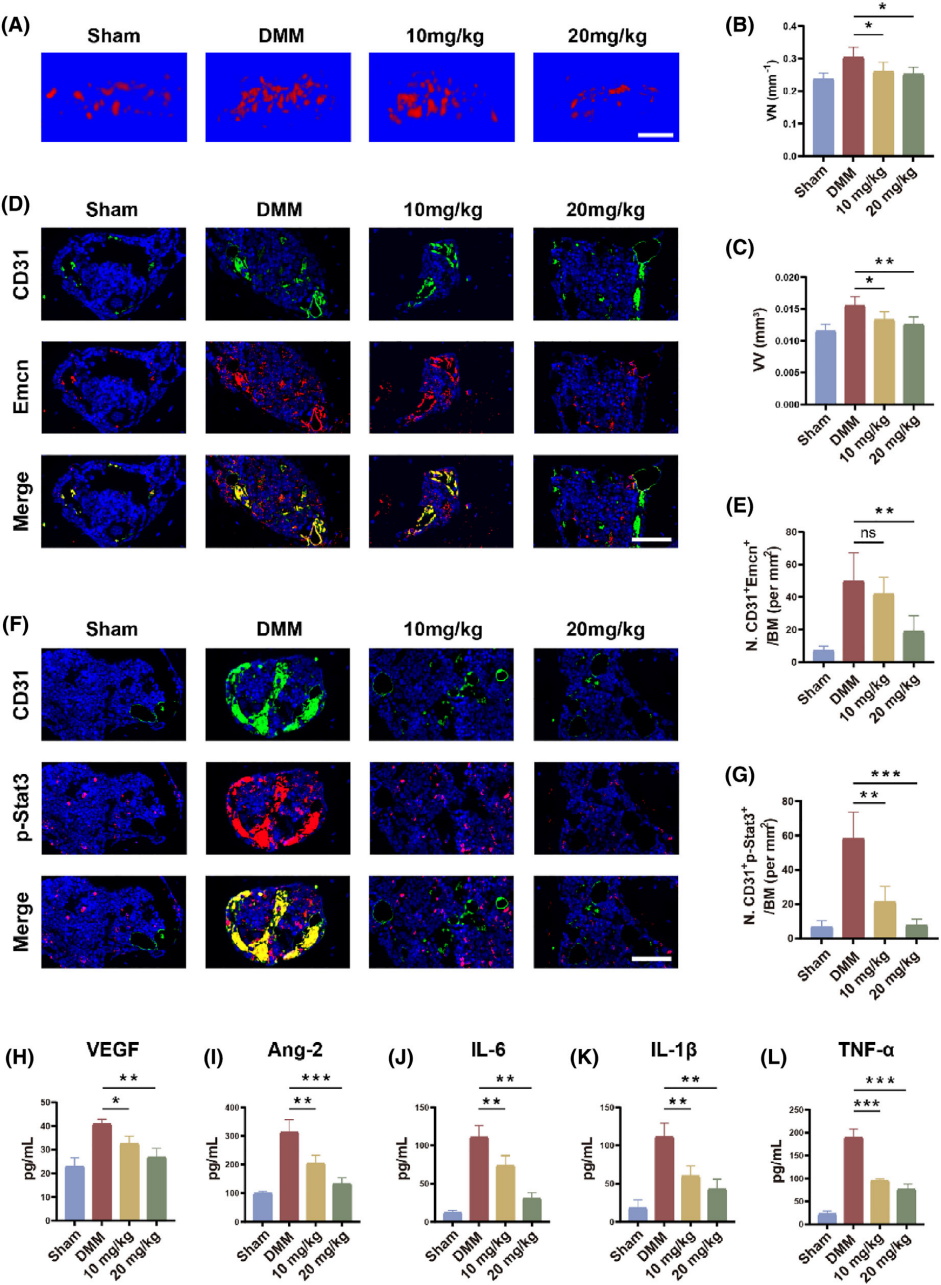
Blocking Stat3 inhibits H‐type vessel neogenesis under OA inflammation. (A) 3D reconstruction for the sagittal plane of the medial subchondral bone blood vessels of the tibia by Micro‐CT at 8 weeks. Scale bar, 500 μm. (B, C) Quantitative analysis of vessel number (VN) (B) and vessel volume (VV) (C) in four groups. (D, E) Representative immunofluorescence pictures (D) and quantitative analysis (E) of CD31 (green) and Emcn (red) positive cells. Scale bar, 50 μm. (F, G) Representative immunofluorescence pictures (F) and quantification (G) of CD31 (green) and p‐Stat3 (red) positive cells. Scale bar, 50 μm. (H–L) The concentrations of VEGF (H), angiopoietin‐2 (I), IL‐6 (J), IL‐1β (K) and TNF‐α (L) in serum samples. All quantified data are presented with mean ± SD. The significance is represented as *p < 0.05, **p < 0.01 and ***p < 0.001.

To further determine whether OA subchondral bone angiogenesis is dependent on Stat3 activation, we investigated the impact of Stattic against abnormal growth of subchondral bone vessels by angiography. The late OA produced a large number of neovascularization, and blocking Stat3 significantly downregulated vascularization of the medial tibial plateau (Figure 6A–C). Similarly, CD31 and Emcn co‐labelled H‐type vessels were significantly reduced (Figure 6D,E). Relative to 10 mg/kg, Stattic at 20 mg/kg demonstrated greater H‐type vessel suppression, almost restoring it to natural levels. In Figure 6F,G, the DMM surgery led to massive activation of Stat3 in H‐type vessels, which promoted neovascularization. After 8 weeks of intraperitoneal injection of 20 mg/kg Stattic, the subchondral bone H‐type vessel generation was dramatically relieved and the expression of p‐Stat3 was diminished. Meanwhile, 10 mg/kg Stattic inhibited the phosphorylation of Stat3 but failed to completely inhibit the proliferation of CD31‐positive vessels. In addition, the secretion of serum angiogenic markers was significantly increased in DMM mice. Stattic inhibits the production of VEGF and angiopoietin‐2 in a dose‐dependent way (Figure 6H,I). Inflammatory factor secretion in the OA microenvironment was also downregulated as Stat3 was blocked (Figure 6J–L). This indicates an alleviated inflammation due to less angiogenesis in DMM mice. In conclusion, Stattic downregulated H‐type vessel angiogenesis by blocking Stat3 activation, while decreasing angiogenic markers and inflammatory markers in OA microenvironment.

### 3.7 Blocking Stat3 reverses angiogenesis‐related bone remodelling and cartilage degeneration

**FIGURE 7:**
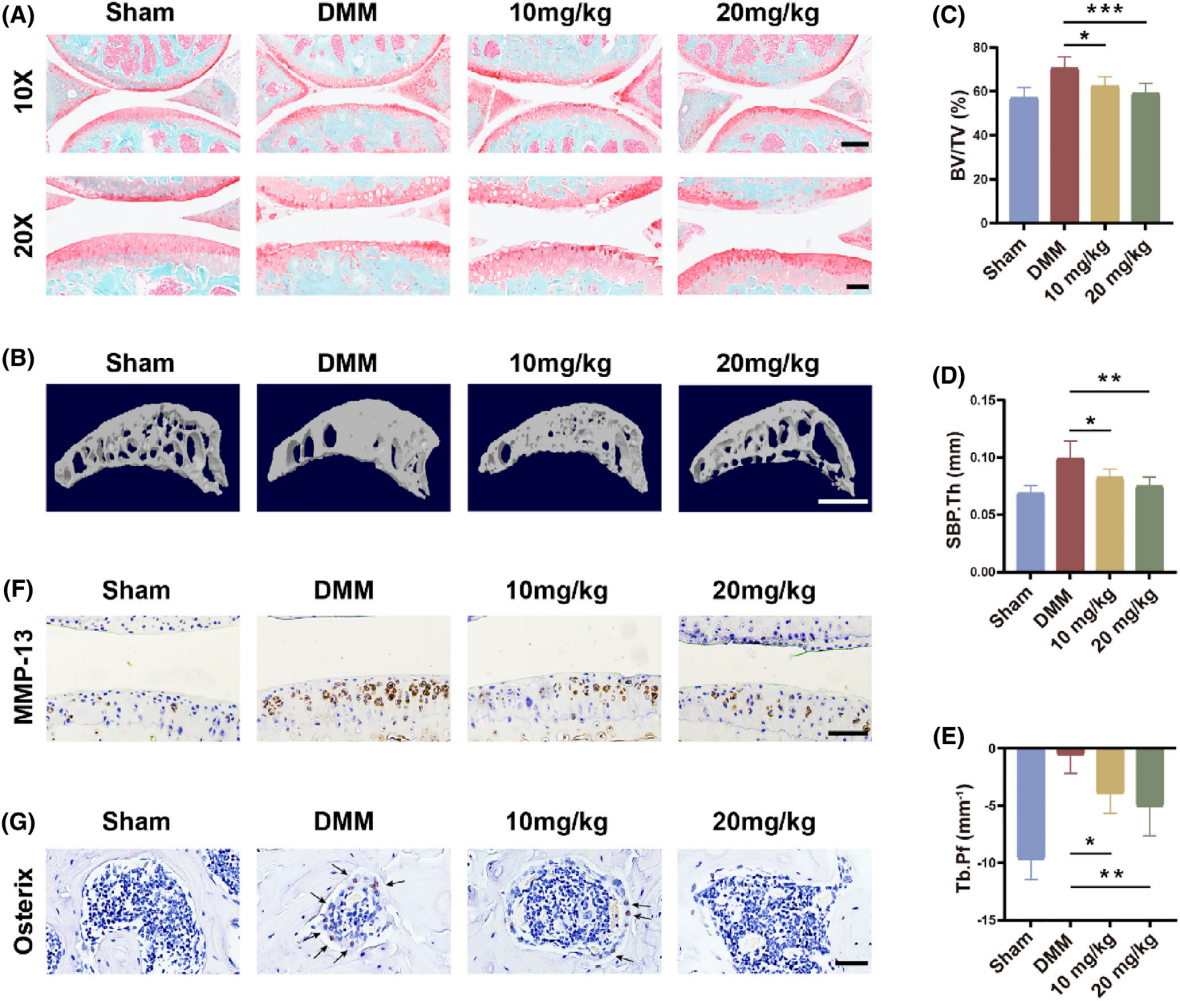
Blocking Stat3 reverses angiogenesis‐related bone remodelling and cartilage degeneration. (A) S&F staining after 8 weeks of DMM modelling. Scale bar, 250 μm (10×). Scale bar, 100 μm (20×). (B) 3D reconstruction for medial tibial plateau subchondral bone sagittal plane 8 weeks after DMM. Scale bar, 500 μm. (C–E) Quantification of subchondral bone volume/tissue volume (BV/TV) (C), subchondral bone plate thickness (SBP.Th) (D) and trabecular separation (Tb.Pf) (E) in the medial tibial plateau by Micro‐CT. (F) Representative immunohistochemical staining of MMP‐13 in articular cartilage. Scale bar, 100 μm. (G) Representative immunohistochemical staining of osterix in subchondral bone. Scale bar, 50 μm. All quantified data are presented with mean ± SD. The significance is represented as *p < 0.05, **p < 0.01 and ***p < 0.001.

Based on the findings above, we evaluated the recovery of articular cartilage in DMM mice by S&F staining. During the 8 weeks of free movement, OA mice developed severe wear and tear of the articular cartilage, with almost distinguishable loss of tibial lateral cartilage (Figure 7A). Relative to the DMM group, Stattic at 20 mg/kg showed superior efficacy to 10 mg/kg, preserving more proteoglycan and hyaline cartilage layer thickness. MicroCT 3D reconstruction showed that Stattic significantly reduced osteophytes, as evidenced by a decrease in BV/TV, SBP.Th, and Tb.Pf (Figure 7B‐E). Similarly, the number and volume of osteophytes at the surgical joint in mice were reduced (Figure S14). This reflected the remission of DMM‐induced hyperosteogeny by blocking Stat3. Immunohistochemistry showed a decrease in MMP‐13 positive cells after inhibition of Stat3, which was more significant in the 20 kg/mg Stattic treatment group (Figures 7F and S15A). In addition, we observed upregulation of the bone progenitor cell marker osterix in the subchondral bone marrow of DMM mice, implying more osteogenesis (Figure 7G). The rise of osterix‐positive cell numbers was reversed by adding Stattic (Figure S15B). It indicates that subsequent subchondral bone remodelling and cartilage lesions were alleviated when angiogenesis was inhibited in OA. In conclusion, our exploration of Stat3 inhibition in vivo suggests that Stat3 could be an effective target for OA therapy.

## 4 DISCUSSION

To date, the incipient pathological changes of OA remain vague, but it is definite that a variety of pathological changes associated with OA may combine to exacerbate the deterioration of OA, including cartilage degeneration, subchondral osteosclerosis, angiogenesis, and nerve growth.^31^, ^32^, ^33^ The OA microenvironment involves chondrocytes, osteocytes, vascular endothelial cells and nerve cells that respond accordingly to chronic inflammation.^34^, ^35^, ^36^ If the drivers of cartilage matrix loss cannot be identified, intercellular communication and feedback effects in the inflammatory microenvironment will promote further deterioration of OA. Therefore, finding and defining joint injury drivers will be a breakthrough solution. Lately, numerous studies suggested that subchondral bone remodelling occurs prior to cartilage injury due to altered joint stress distribution and mechanical structural imbalance.^37^, ^38^, ^39^, ^40^ Simultaneously, a significant distribution of neovascularization in the subchondral bone after OA injury was observed in both human and animal samples.^30^ At the same time, the nociceptive nerve grows into the articular cavity along with the new blood vessels.^11^, ^14^ It may even penetrate the tibial plateau to reach the joint cavity, causing severe pain to the patient.^41^ Similar pathological changes were observed in clinical samples with neovascular invasion along nerve fibres breaking through the tidal markers and in the bony bulge of OA.^41^ Nerve growth factor (NGF) was shown to facilitate the growth of blood vessels and nerves in OA subchondral bone, thereby connecting angiogenesis and joint pain in OA.^42^ The abnormal neovascularization is thought to cause elevated bone density and microstructural deterioration, ultimately resulting in severe cartilage destruction. However, subchondral bone angiogenesis is a multidimensional process with mechanisms possibly involving the co‐regulation of multiple cytokines and signalling pathways.^43^ Here, we found a significant increase in Stat3 activation in OA subchondral bone vessels. Endothelial Stat3 activation exacerbates OA by inducing angiogenesis, BMSCs osteogenesis and chondrocyte damage.

Stat3 is involved in the intracellular transduction of multiple cytokines such as IL‐6, VEGF and PDGF.^44^, ^45^, ^46^ Latourte et al. examined the protective impact of systemic inhibition of Stat3 and found that blockade of chondrocyte Stat3 signalling could achieve OA reversal.^22^ This is probably due to the fact that Stattic downregulates the sensitivity of chondrocytes to cytokines such as IL‐6 and VEGF. Notably, we found that endothelial Stat3 absence would weaken the ability to induce catabolic genes (MMP‐3, MMP‐13) in chondrocytes, thereby restoring the total amount of cartilage extracellular matrix proteoglycans. This implies that endothelial Stat3 activation may be a bridge between angiogenesis and cartilage injury in OA.

Previous study showed that Stat3 activation would promote the growth of blood vessels within the tumour hypoxic environment to provide more nutrients and oxygen.^29^, ^47^, ^48^, ^49^ Many Stat3 inhibitors have proven to be curative in clinical trials.^50^ Likewise, as a hypoxic microenvironment, trace amounts of vessels should provide adequate nutrients without affecting the normal structure of the subchondral bone.^51^ When endothelial Stat3 is activated, more endothelial cells migration and angiogenesis means higher oxygen levels and nutrients in the surrounding tissue. It will completely reverse the components of the OA microenvironment to promote disease progression. Here, we activated Stat3 by transient incubation with IL‐6 to mimic ECs within the OA microenvironment. Using small molecule inhibitors and genetically engineered plasmids, we identified that normal activation and expression of Stat3 is essential for the proliferation, migration and angiogenesis of ECs in an inflammatory environment. Similarly, the in vivo experiments proved that blocking Stat3 signalling facilitated to restrain the abnormal proliferation of H‐type vessels in OA. However, targeted inhibition of endothelial Stat3 is not achieved due to the drawbacks of systemic administration. Knockdown of endothelial Stat3 in mice or targeted delivery of Stattic to ECs would provide more powerful evidences. On the other hand, due to the regulatory effect of Stat3 inhibition on different cells in the OA microenvironment, it is implied that non‐targeted Stat3 inhibition would synergistically enhance its efficacy. Blocking Stat3 is expected to be a promising multi‐targeted therapeutic option for OA.

To date, connection and regulatory mechanisms linking subchondral bone remodelling and cartilage damage are still unclear. In addition, Stat3 plays an important role in regulating macrophage polarization in an inflammatory environment. When activated, the Stat3 signalling pathway can protect the M2 phenotype of macrophages in the presence of IFN‐γ, thereby greatly alleviating OA microenvironmental inflammation.^52^ Meanwhile, Stat3 was activated by IL‐22 to induce the osteoclast generation.^53^ Phosphorylation modification of Stat3 at the Tyr705 site induces TRAP‐positive osteoclast fusion.^53^ This implies that Stat3 is involved in the regulation of the early bone resorption process in OA. In addition, as the patient's disease advances, elevated inflammatory factors in body fluids will activate the Stat3 signalling pathway in osteoblasts. With the activation of Stat3 signalling, osteoblasts exhibit stronger ALP activity and mineralized calcium formation.^54^ All of the above evidence implies that Stat3 exhibits an irreplaceable role in the whole OA microenvironment. In previous work, endothelial PDGF‐BB/PDGFR‐β pathway was proved to facilitate OA by reinforcing angiogenesis‐related bone formation.^25^, ^55^, ^56^ The endothelial Stat3 activation by pro‐osteoclasts was also observed in our study, suggesting that Stat3 is a pivotal regulatory protein coupling multiple cells in the OA microenvironment. More interestingly, we found that Stat3‐activated ECs significantly promoted BMSCs osteogenic differentiation and triggered chondrocyte inflammation. Thus, mediated by endothelial Stat3 activation, osteoclast‐mediated bone resorption gradually evolves into severe subchondral bone remodelling and cartilage damage.

**FIGURE 8:**
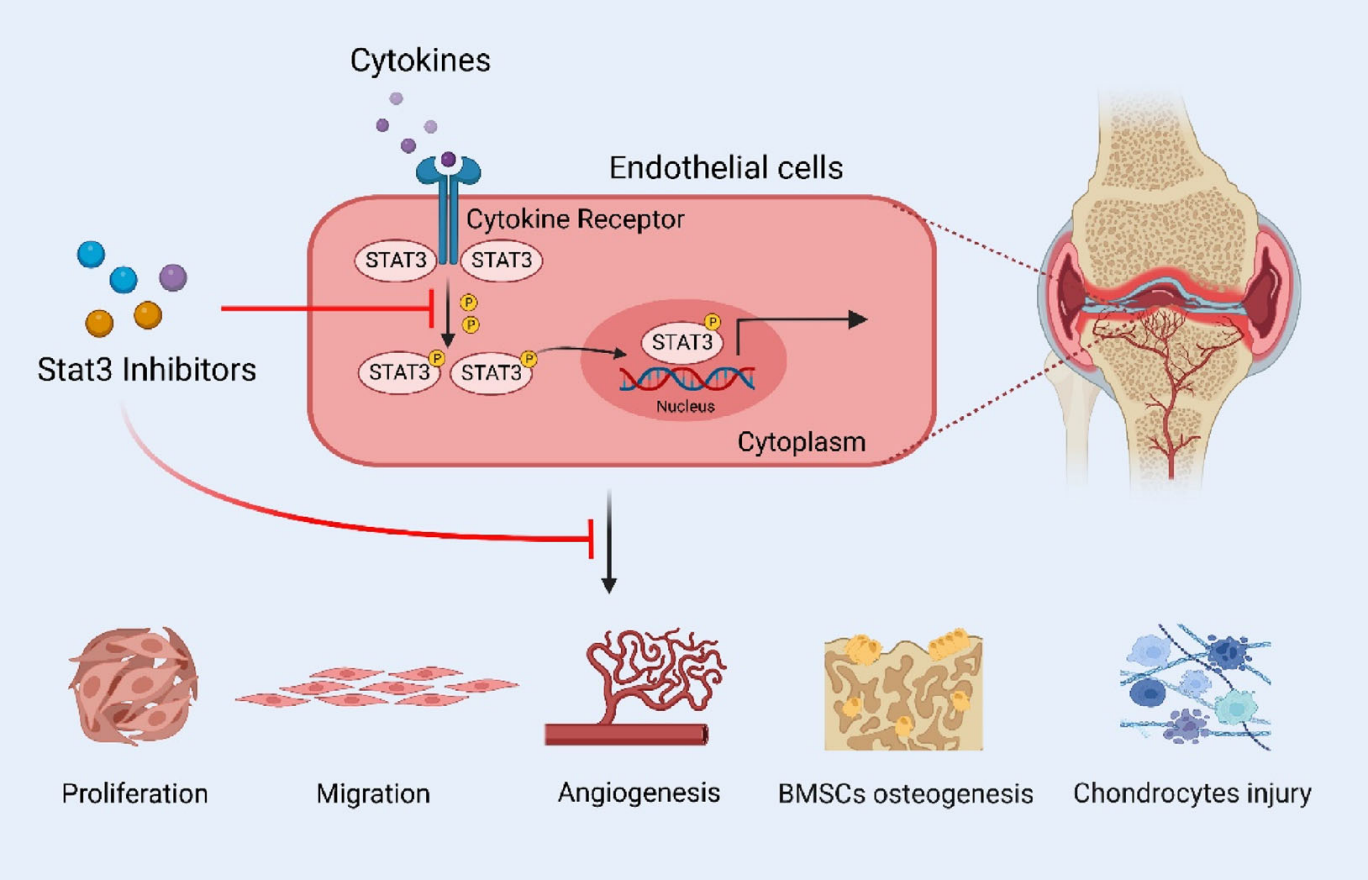
Schematic diagram of endothelial Stat3 regulation on OA progression.

In summary, we revealed endothelial Stat3 activation as a pivotal factor in promoting OA progression (Figure 8). Endothelial Stat3 was activated by multiple cytokines in the OA microenvironment to enhance the proliferation, migration and angiogenesis. Phosphorylation inhibition or reduced expression of endothelial Stat3 attenuated OA‐induced angiogenesis. Endothelial Stat3 activation possessed stronger capacity for osteogenic induction and cartilage destruction. Systemic Stat3 blockade alleviated subchondral bone H‐type vessel formation and abnormal bone remodelling in DMM mice. To summarize, our data provide new mechanistic insights into how endothelial Stat3 regulate the subchondral bone microenvironment during OA development. Stat3 is hence a promising new target for OA treatment.

## AUTHOR CONTRIBUTIONS

Jiadong Li, Xinru Liu, and Guangfeng Li conceived the study and designed the experiments. Jiadong Li, Xinru Liu, and Guangfeng Li performed the experiments, acquired the data and analysed the results. Yuyuan Gu, Kun Zhang, Fuming Shen, Xiang Wu, Yingying Jiang and Qin Zhang provided technical consultation and contributed to data interpretation. Jiadong Li, Fengjin Zhou, Ke Xu and Jiacan Su prepared the article. Wencai Zhang revised the manuscript and supplementary information in the process of revision. Jiadong Li, Wencai Zhang, Xinru Liu, and Guangfeng Li contributed equally to this work.

## CONFLICT OF INTEREST STATEMENT

All authors declare no conflict of interest.

## Supporting information


**Data S1:** Supporting Information

## Data Availability

The data that support the findings of this study are available from the corresponding author upon reasonable request.
