# Supplementary material for: Endothelial Stat3 activation promotes osteoarthritis development
Source: Cell Prolif. 2023 Jun 13;56(12):e13518. doi: 10.1111/cpr.13518 (PMC10693181; doi:10.1111/cpr.13518)
Supplement: Supplementary file 1 — Data S1: Supporting Information [file CPR-56-e13518-s001.docx]

**Endothelial Stat3 activation promotes osteoarthritis development**

**SUPPLEMENTARY MATERIALS**

**Figure S1**


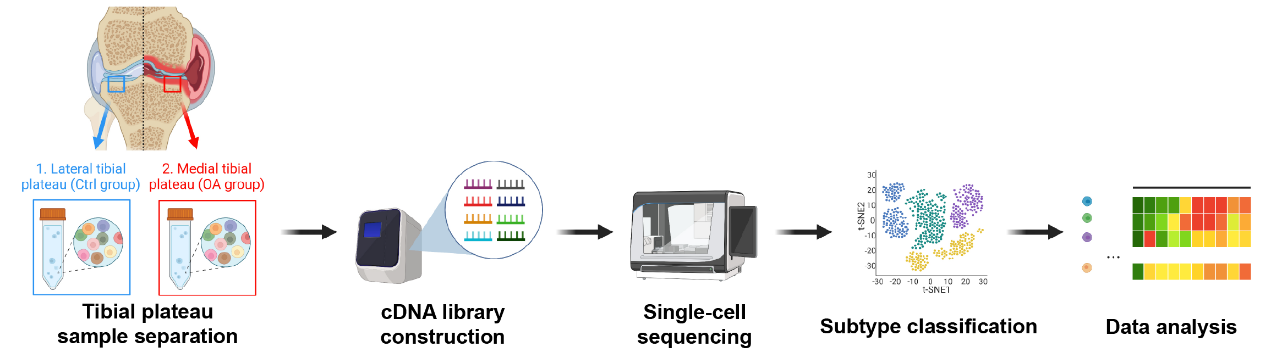


**Figure S1** Schematic diagram of single cell sequencing process.

**Figure S2**


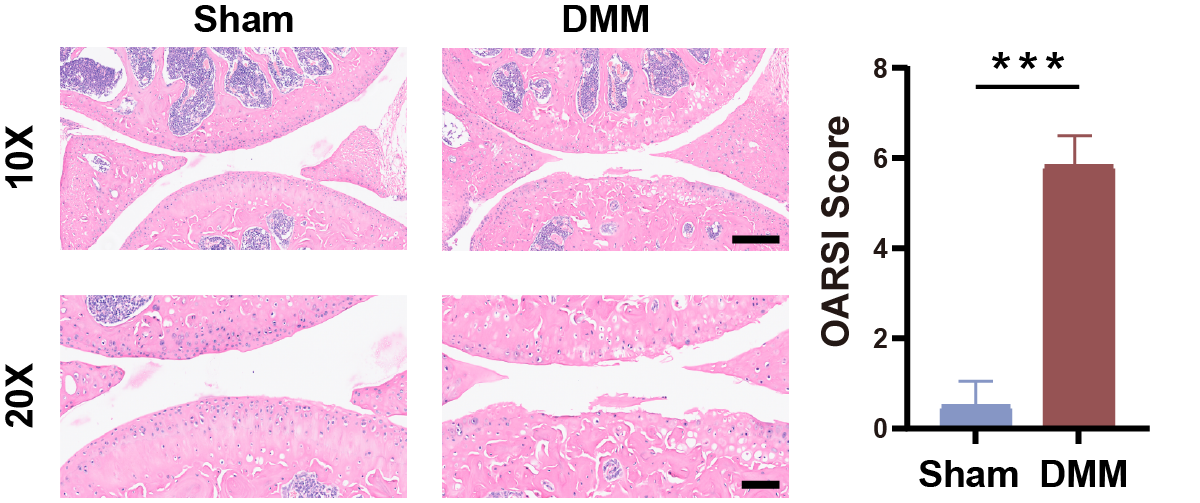


**Figure S2** DMM surgery caused severe joint damage in mice within 8 weeks. S&F staining (left) and OARSI scores (right) at 8 weeks after DMM modeling. Scale bar = 250 μm (10X). Scale bar = 100 μm (20X). All quantified data are presented with mean ± SD. The significance is represented as *p < 0.05, **p < 0.01 and ***p < 0.001.

**Figure S3**


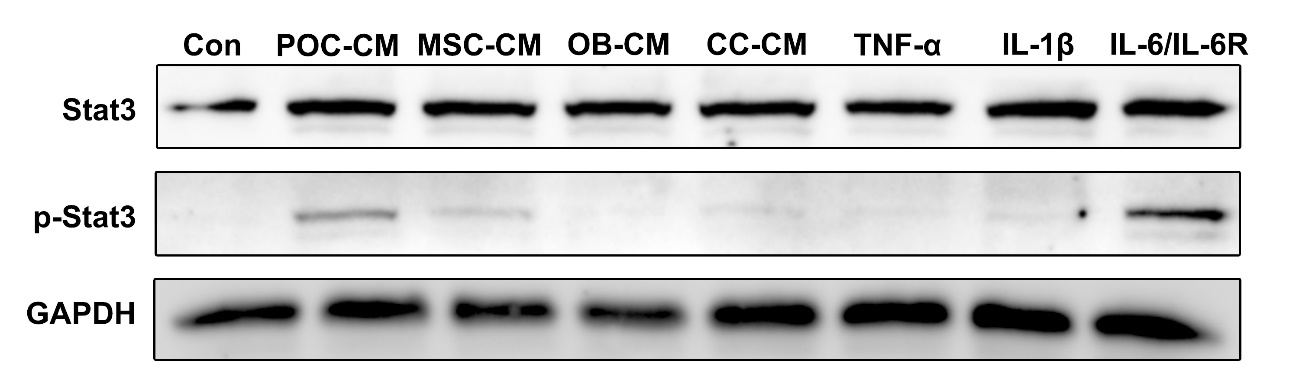


**Figure S3** Activation of endothelial Stat3 by different cell conditioned media. ECs were incubated with different conditioned media for 30 min respectively and then total proteins were extracted. Pre-osteoblastic cells conditioned media: POC-CM. Mesenchymal stem cells conditioned media: MSC-CM. Osteoblast conditioned media: OB-CM. Chondrocyte conditioned media: CC-CM. Inflammatory factors: 10ng/mL TNF-α, 10ng/mL IL-1β, 100ng/mL IL-6 + 100ng/mL IL-6R.

**Figure S4**


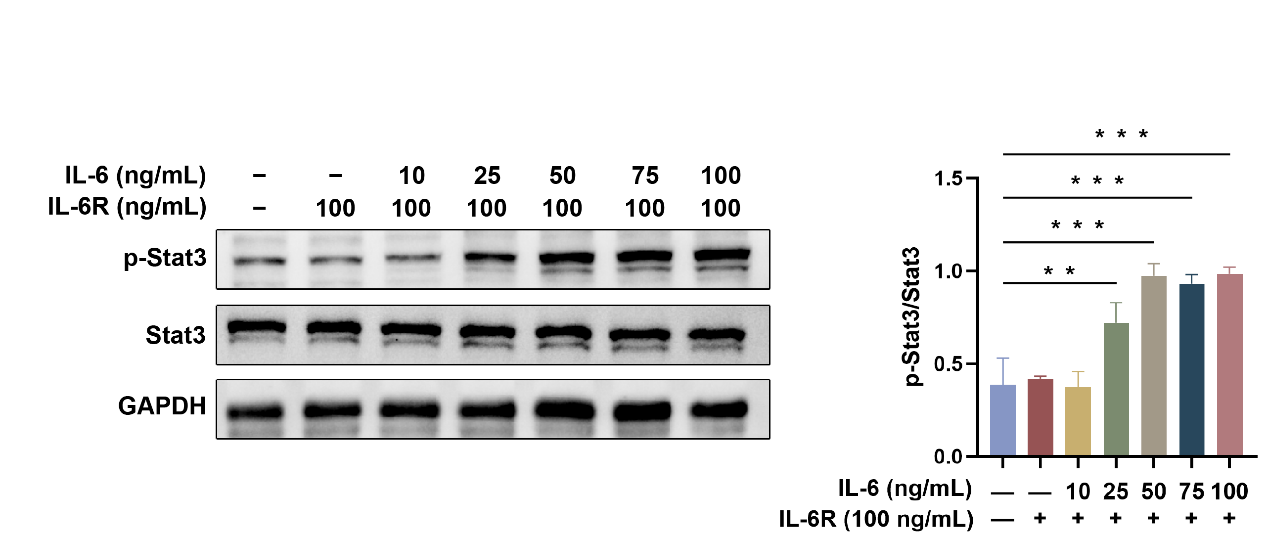


**Figure S4** IL-6 activates endothelial Stat3 signaling in a concentration-dependent manner. Western blot assay (left) for Stat3 activation and quantitative analysis (right). All quantified data are presented with mean ± SD. The significance is represented as *p < 0.05, **p < 0.01 and ***p < 0.001.

**Figure S5**


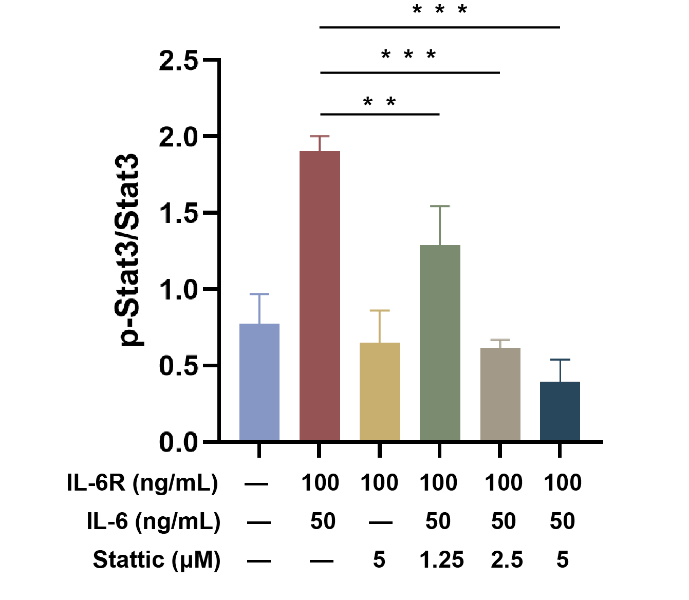


**Figure S5** Western blot quantitative analysis of Stattic inhibition to endothelial Stat3 activation. Stattic and IL-6/IL-6R were co-incubated with ECs for 30 min and then total protein was extracted. All quantified data are presented with mean ± SD. The significance is represented as *p < 0.05, **p < 0.01 and ***p < 0.001.

**Figure S6**


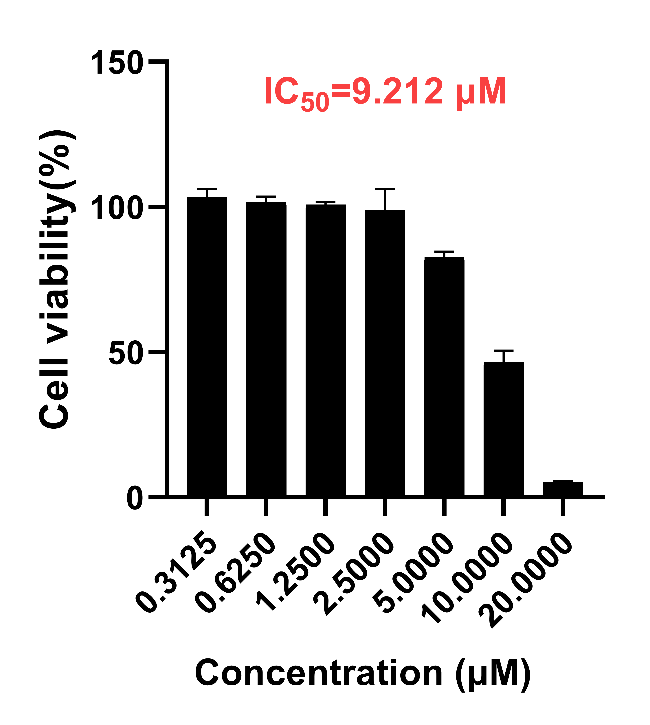


**Figure S6** Cytotoxicity assay and IC_50_ of different concentrations of Stattic. Stattic was incubated with ECs in an incubator at 37°C for 48h and then cell viability was tested by the CCK-8 kit. All quantified data are presented with mean ± SD.

**Figure S7**


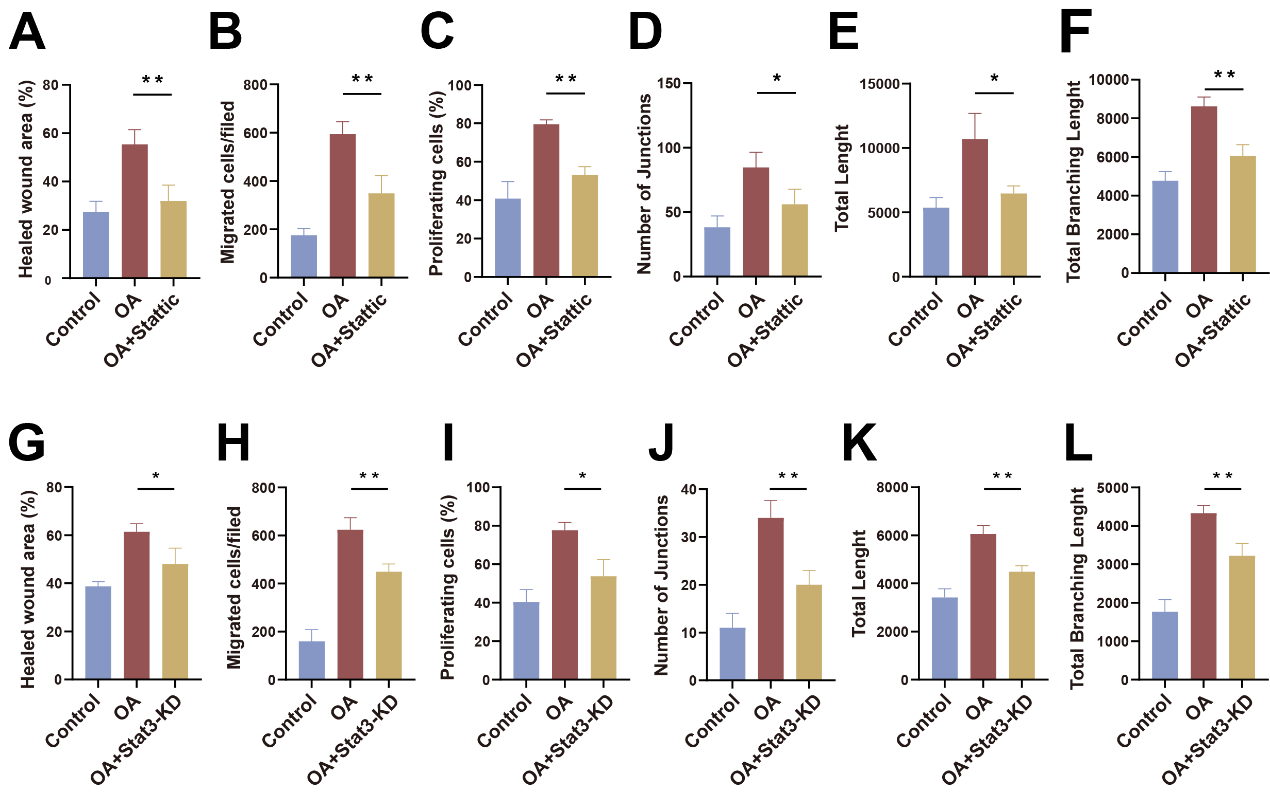


**Figure S7** Inhibiting endothelial Stat3 activation alleviates OA-induced angiogenesis. (A) Quantitative analysis of wound healing areas in different groups of ECs. (B) Quantification of migrating cells with crystalline violet staining in bright fields. (C) Quantification of EdU fluorescence-positive proliferating ECs. (D-F) Number of junctions (D), total length (E) and total branching length (F) of tubes formed by ECs on the matrigel in 6 hours. (G) Quantitative analysis of wound healing areas in different groups of ECs. (H) Quantification of migrating cells with crystalline violet staining in bright fields. (I) Quantification of EdU fluorescence-positive proliferating ECs. (J-L) Number of junctions (J), total length (K) and total branching length (L) of tubes formed by ECs on the matrigel in 6 hours. All quantified data are presented with mean ± SD. The significance is represented as *p < 0.05, **p < 0.01 and ***p < 0.001.

**Figure S8**


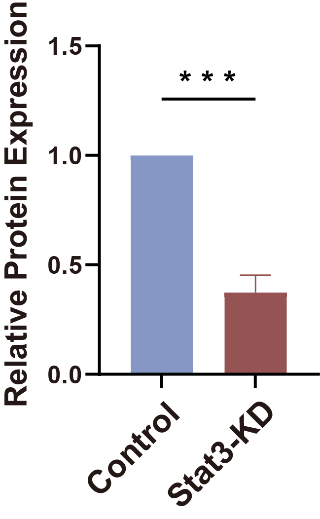


**Figure S8** Quantitative analysis of ECs Stat3 knockdown with control plasmid and Stat3-KD plasmid. All quantified data are presented with mean ± SD. The significance is represented as *p < 0.05, **p < 0.01 and ***p < 0.001.

**Figure S9**


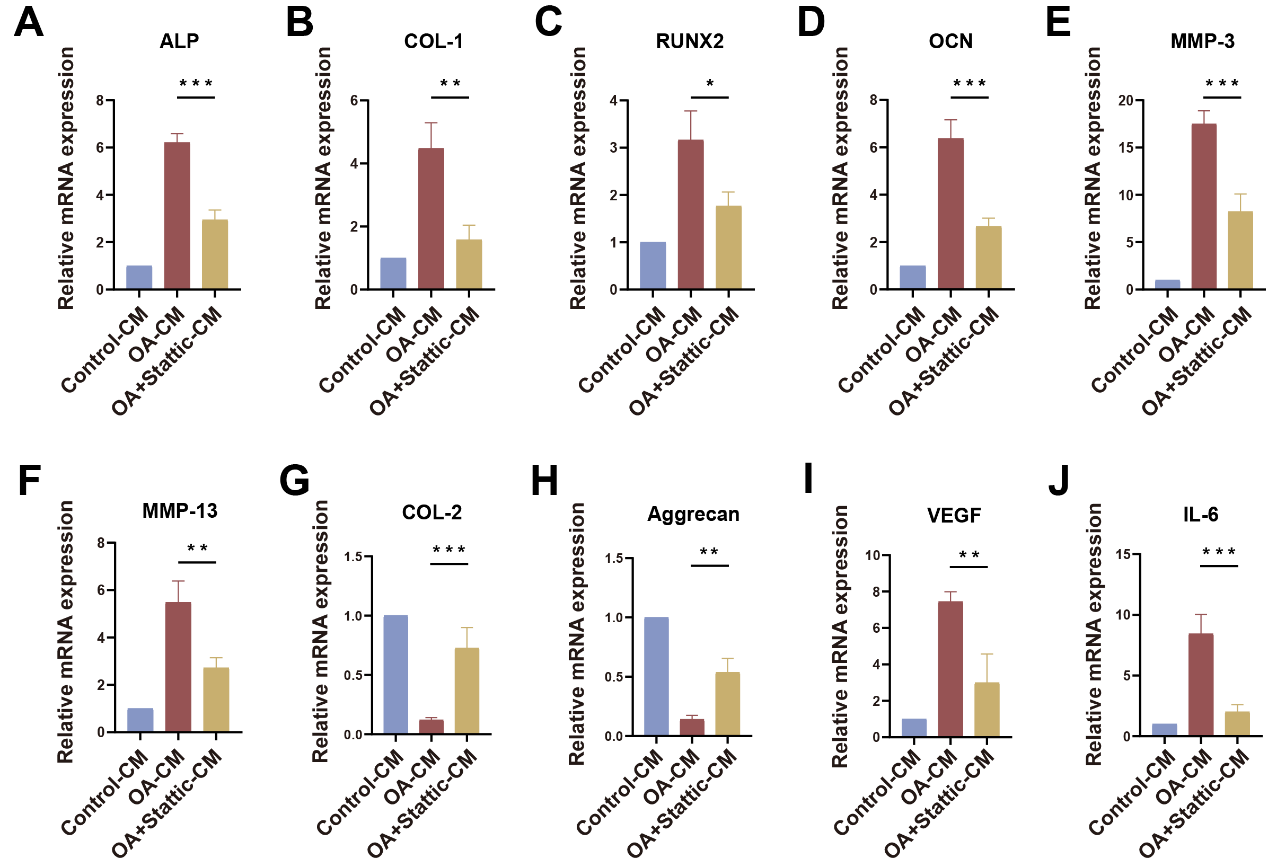


**Figure S9** Inhibition of endothelial Stat3 phosphorylation suppresses BMSCs osteogenic and chondrocyte injury. (A-D) qRT-PCR results of osteogenic marker (ALP, COL-1, RUNX2, OCN) expression in BMSCs after incubation with different conditioned media for 7 days. (E-J) qRT-PCR results of chondrocyte catabolic markers (MMP-3, MMP-13), anabolic markers (COL-2, Aggrecan), and inflammatory markers (VEGF, IL-6) expression after incubation with different conditioned media for 48h. All quantified data are presented with mean ± SD. The significance is represented as *p < 0.05, **p < 0.01 and ***p < 0.001.

**Figure S10**


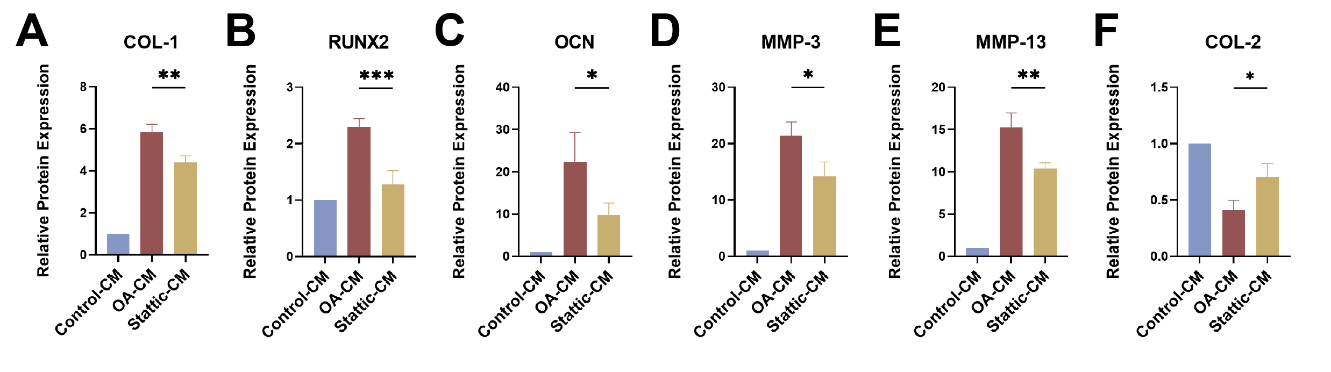


**Figure S10** Western blot quantitative analysis of osteogenic markers (COL-1, RUNX2, OCN) after 7 days incubation and chondrocyte markers (MMP-3, MMP-13, COL-2) after 48h incubation with different conditioned medium (Control-CM, OA-CM, Stattic-CM). All quantified data are presented with mean ± SD. The significance is represented as *p < 0.05, **p < 0.01 and ***p < 0.001.

**Figure S11**


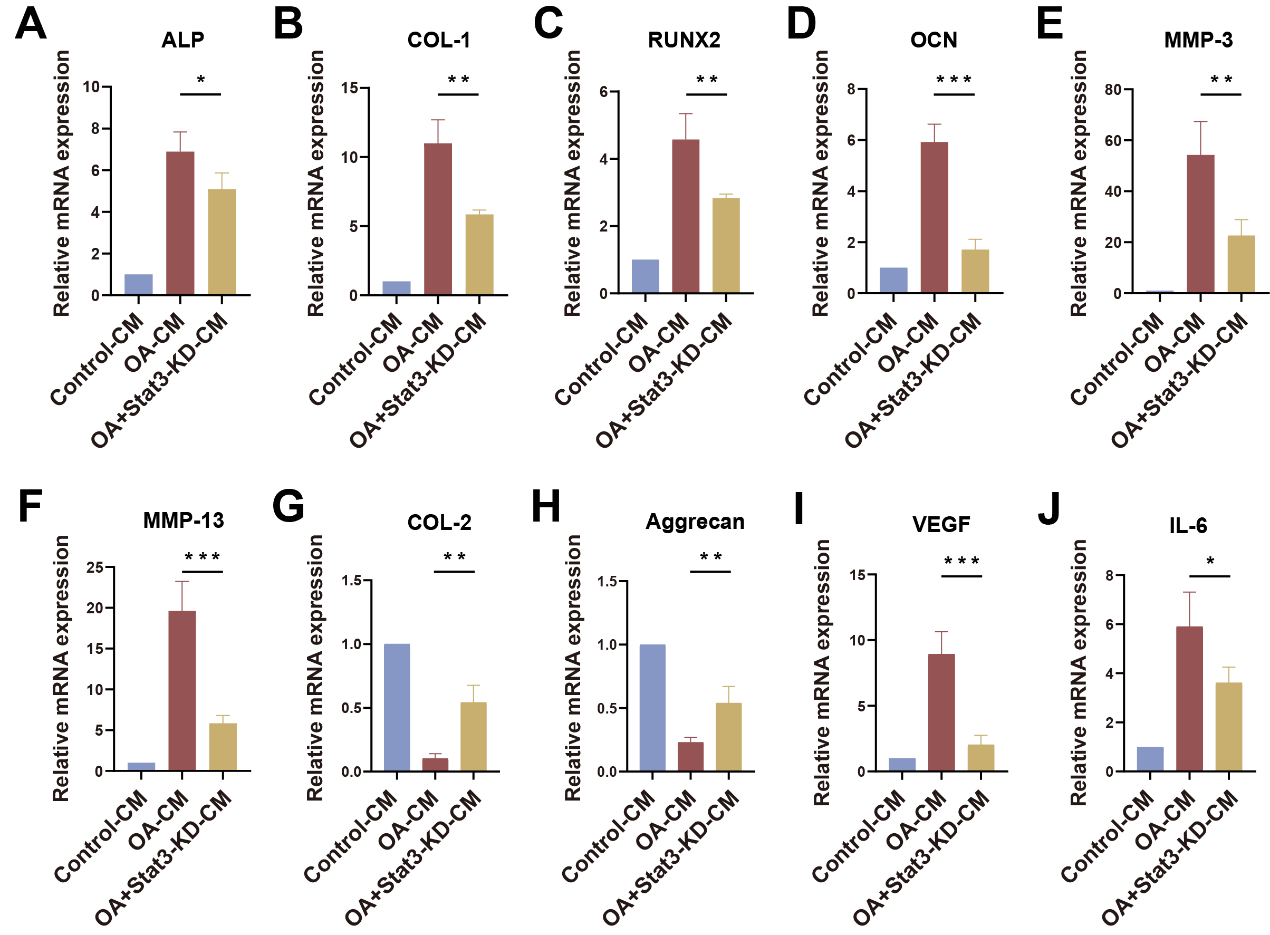


**Figure S11** Endothelial Stat3 knockdown inhibits BMSCs osteogenesis and chondrocyte injury. (A-D) qRT-PCR results of osteogenic marker (ALP, COL-1, RUNX2, OCN) expression in BMSCs after incubation with different conditioned media for 7 days. (E-J) qRT-PCR results of chondrocyte catabolic markers (MMP-3, MMP-13), anabolic markers (COL-2, Aggrecan), and inflammatory markers (VEGF, IL-6) expression after incubation with different conditioned media for 48h. All quantified data are presented with mean ± SD. The significance is represented as *p < 0.05, **p < 0.01 and ***p < 0.001.

**Figure S12**


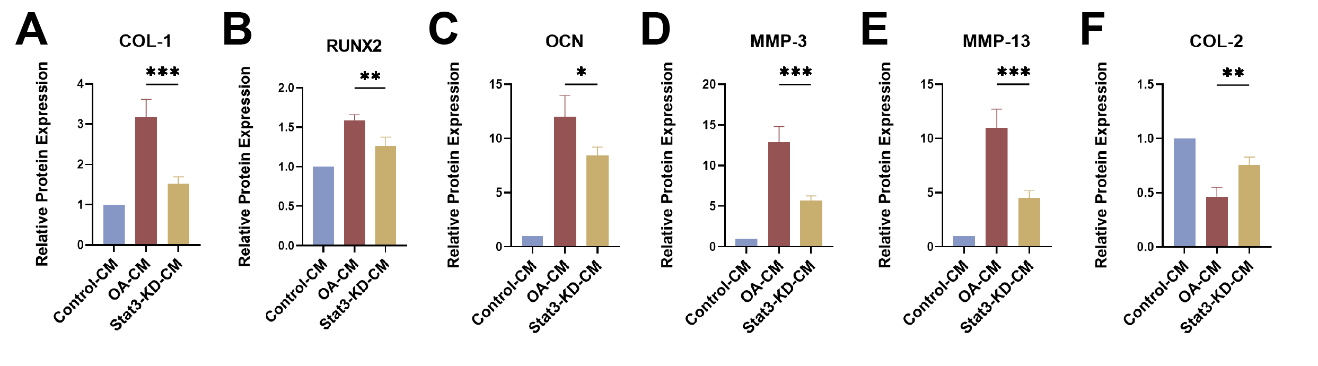


**Figure S12** Western blot quantitative analysis of osteogenic markers (COL-1, RUNX2, OCN) after 7 days incubation and chondrocyte markers (MMP-3, MMP-13, COL-2) after 48h incubation with different conditioned medium (Control-CM, OA-CM, Stat3-KD-CM). All quantified data are presented with mean ± SD. The significance is represented as *p < 0.05, **p < 0.01 and ***p < 0.001.

**Figure S13**


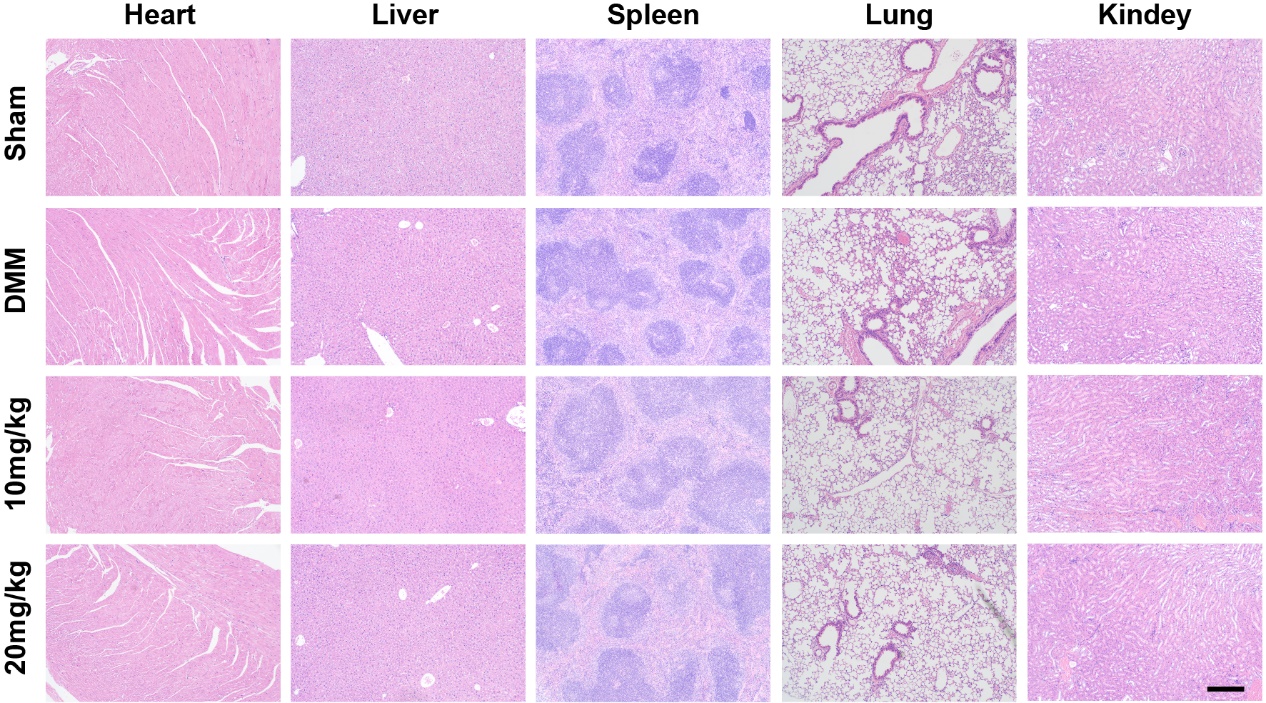


**Figure S13** Toxicity assessment of Stattic in vivo. H&E staining of major organ sections. Scale bar = 200 μm.

**Figure S14**


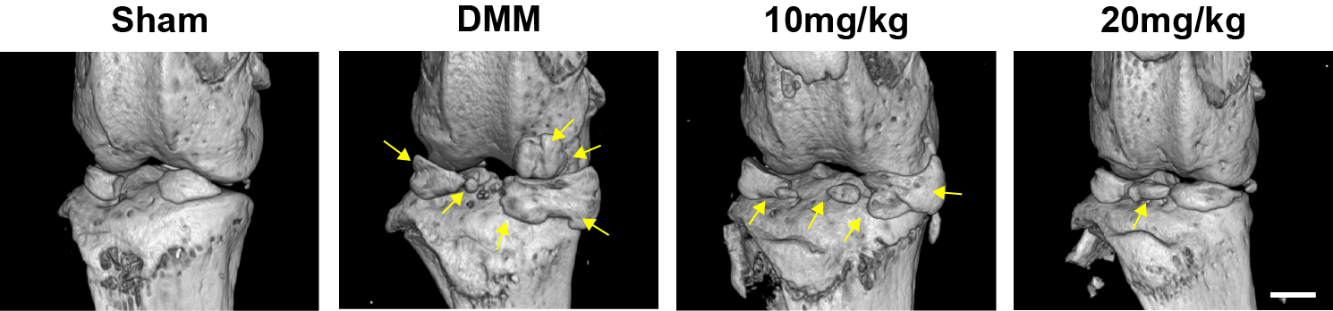


**Figure S14** Stattic reverses osteophyte formation in the joint. Representative three-dimensional images of articular cartilage reconstructed by Micro-CT. Scale bar = 500 μm.

**Figure S15**


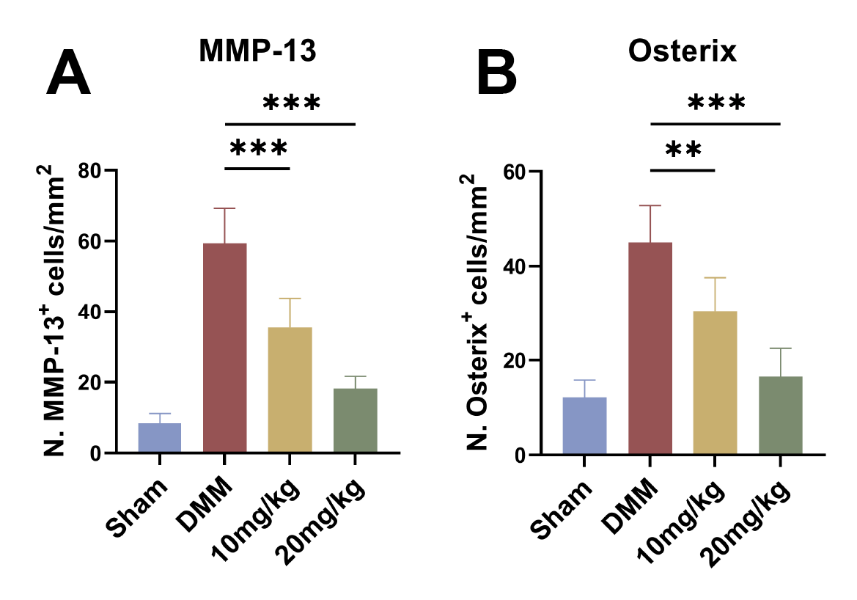


**Figure S15** IHC quantitative analysis of MMP-13 and Osterix. All quantified data are presented with mean ± SD. The significance is represented as *p < 0.05, **p < 0.01 and ***p < 0.001.
